# A Systematic Review on Marine Algae-Derived Fucoxanthin: An Update of Pharmacological Insights

**DOI:** 10.3390/md20050279

**Published:** 2022-04-22

**Authors:** Md. Mohibbullah, Md. Nazmul Haque, Abdullah Al Mamun Sohag, Md. Tahmeed Hossain, Md. Sarwar Zahan, Md. Jamal Uddin, Md. Abdul Hannan, Il Soo Moon, Jae-Suk Choi

**Affiliations:** 1 Department of Fishing and Post Harvest Technology, Sher-e-Bangla Agricultural University, Sher-e-Bangla Nagar, Dhaka 1207, Bangladesh; mmohib.fpht@sau.edu.bd; 2 Seafood Research Center, Silla University, #605, Advanced Seafood Processing Complex, Wonyang-ro, Amnam-dong, Seo-gu, Busan 49277, Korea; 3 Department of Food Biotechnology, Division of Bioindustry, College of Medical and Life Sciences, Silla University, Busan 46958, Korea; 4 Department of Anatomy, College of Medicine, Dongguk University, Gyeongju 38066, Korea; habib.332@gmail.com (M.N.H.); moonis@dongguk.ac.kr (I.S.M.); 5 Department of Fisheries Biology and Genetics, Patuakhali Science and Technology University, Patuakhali 8602, Bangladesh; 6 Department of Biochemistry and Molecular Biology, Bangladesh Agricultural University, Mymensingh 2202, Bangladesh; sohag2010bmb.sust@gmail.com (A.A.M.S.); tahmeed.hossain@bau.edu.bd (M.T.H.); hannanbmb@bau.edu.bd (M.A.H.); 7 ABEx Bio-Research Center, East Azampur, Dhaka 1230, Bangladesh; mszahan@hotmail.com (M.S.Z.); hasan800920@gmail.com (M.J.U.)

**Keywords:** fucoxanthin, bioactivities, in vitro, in vivo, pharmacokinetics, safety and toxicity

## Abstract

Fucoxanthin, belonging to the xanthophyll class of carotenoids, is a natural antioxidant pigment of marine algae, including brown macroalgae and diatoms. It represents 10% of the total carotenoids in nature. The plethora of scientific evidence supports the potential benefits of nutraceutical and pharmaceutical uses of fucoxanthin for boosting human health and disease management. Due to its unique chemical structure and action as a single compound with multi-targets of health effects, it has attracted mounting attention from the scientific community, resulting in an escalated number of scientific publications from January 2017 to February 2022. Fucoxanthin has remained the most popular option for anti-cancer and anti-tumor activity, followed by protection against inflammatory, oxidative stress-related, nervous system, obesity, hepatic, diabetic, kidney, cardiac, skin, respiratory and microbial diseases, in a variety of model systems. Despite much pharmacological evidence from in vitro and in vivo findings, fucoxanthin in clinical research is still not satisfactory, because only one clinical study on obesity management was reported in the last five years. Additionally, pharmacokinetics, safety, toxicity, functional stability, and clinical perspective of fucoxanthin are substantially addressed. Nevertheless, fucoxanthin and its derivatives are shown to be safe, non-toxic, and readily available upon administration. This review will provide pharmacological insights into fucoxanthin, underlying the diverse molecular mechanisms of health benefits. However, it requires more activity-oriented translational research in humans before it can be used as a multi-target drug.

## 1. Introduction

**Figure 1:**
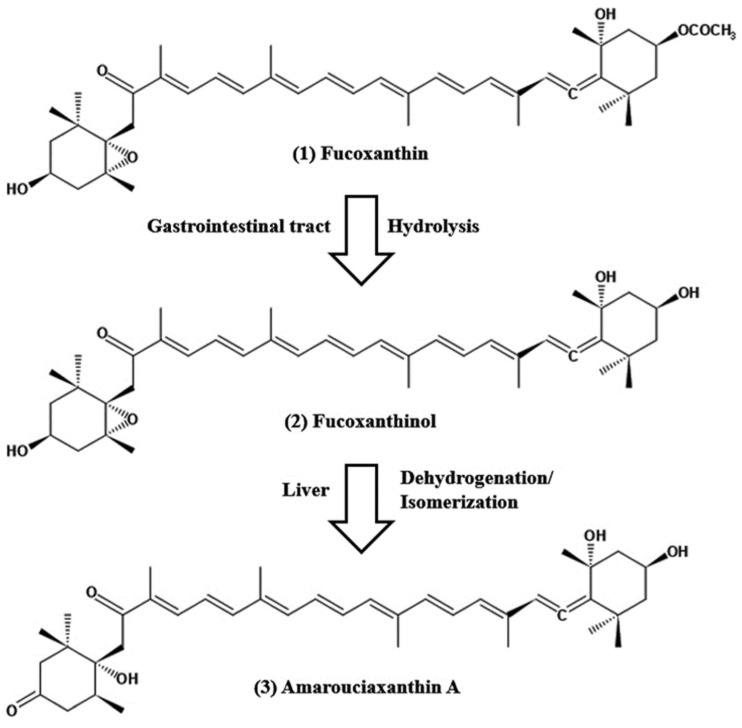
Chemical structure of fucoxanthin and its derivatives, fucoxanthinol by hydrolysis with digestive enzymes, lipase and cholesterol esterase in the gastrointestinal tract and ama-rouciaxanthin A by short chain dehydrogenase/reductase in the liver.

Fucoxanthin is a well-known naturally occurring carotenoid from marine algae, especially brown macroalgae or seaweed and diatoms. It is an orange pigment that contributes to more than 10% of the total available carotenoids in nature [1]. It is essentially associated with chlorophylls a and c and β-carotene, which is functionally involved in the light-harvesting complex of the algae [2]. The diverse health-promoting effects of fucoxanthin are attributed to its unique chemical structure such as an acetyl group, an allenic bond, and a conjugated carbonyl, along with 5,6-monoepoxide (Figure 1). Since fucoxanthin has attracted remarkable attention from the scientific community worldwide as a single compound with multi-potent health effects, the global fucoxanthin market has been dramatically increasing from USD 88 million in 2019 to more than USD 100 million over the following five years, from a report from Global Fucoxanthin Market 2020.

Fucoxanthin is abundant in brown seaweeds that are consumed as dietary supplements and traditional or herbal medicines worldwide, including in Southeast Asian countries and many European countries [3,4]. In East Asia, fucoxanthin-rich seaweeds, genera of Undaria, Laminaria and Sargassum are included in the daily diet and used as herbal medicines to treat various diseases [1]. Researchers are increasingly interested in the pharmacological importance of this naturally occurring fucoxanthin, alongside applications in the food and cosmeceutical industries [5]. Fucoxanthin has strong antioxidant potency, as evidenced by various cell culture models [6,7] and animal studies [8,9], potentially involved in regulating the Nrf2/ARE pathway. Yang and his colleague reported the anti-inflammatory effect of fucoxanthin [10], and their molecular mechanisms of prevention were characterized by the inhibition of NF-κB-related pathways [11]. The anticancer effects of fucoxanthin in the human breast cancer cell line of MDA-MB-231 cells [12], human leukemia cell lines of K562 and TK6 [13] and the mice cancer model [14] are attributed to their regulation of anti-apoptotic, antioxidant and anti-inflammatory pathways, as reported previously. The administration of fucoxanthin to humans significantly reduces the relative body weight in a double-blind placebo-controlled study [15], along with in vitro [16] and in vivo [17] findings. Furthermore, fucoxanthin acts as a neurotrophic factor-like substance, conferring neuroprotection [18] and neurite outgrowth [19] in CNS neurons. In addition, fucoxanthin can ameliorate metabolic [3], hepatic [9], renal [20], cardiovascular [21], bone [22], ocular [23], skin [24], and respiratory [25] diseases, and show antimicrobial potentials [26].

Despite substantial evidence of the pharmacological benefits of fucoxanthin, the successful transformation of preclinical research into clinical subjects is rarely found, which further limits the functional use of this compound at nutraceutical and pharmaceutical levels. Therefore, the authors review the recent scientific literature on fucoxanthin, being focused on pharmacological properties with molecular mechanisms, to facilitate the one step ahead of translational research into human subjects. Additionally, based on the existing literature, pharmacokinetics, safety, toxicity, functional stability, and the clinical perspective of fucoxanthin, which are essential to consider before nutraceutical and pharmaceutical development, are critically highlighted in the latter part to ensure the effective drug delivery for bio-accessibility and bio-functionality after administration.

## 2. Results and Discussion

### 2.1. Current Research Trends on Fucoxanthin

**Figure 2:**
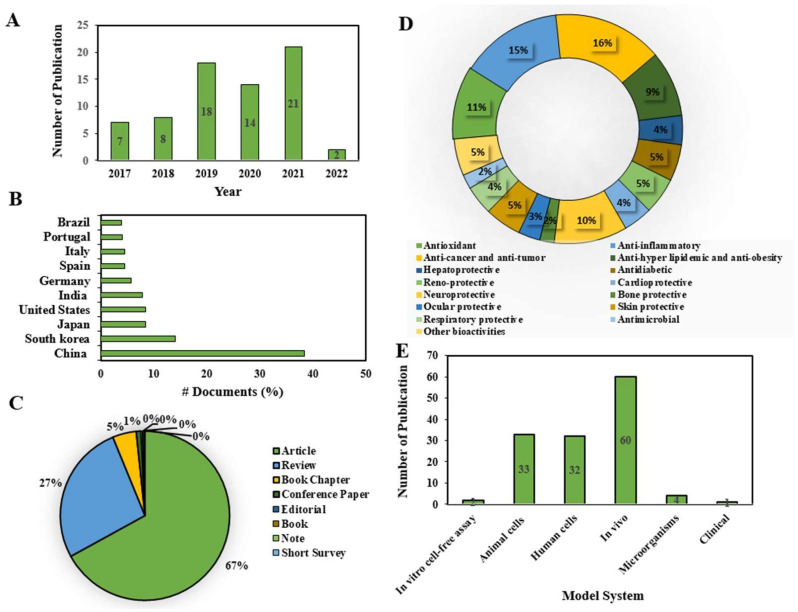
Research trends in fucoxanthin. (**A**) The number of publications per year on the pharmacological properties from 2017 to 2022. (**B**) Countries with the highest number of publications. (**C**) Document-wise publications in percentage, (**D**) Number of publications in percentage according to pharmacological properties. (**E**) Model system-wise publications.

The scientific literature published between January 2017 and February 2022 on the pharmacological properties of fucoxanthin, either used in its purified form or extracted from marine algae, was systematically reviewed. Fucoxanthin was first isolated from the marine brown seaweeds Fucus, Dictyota, and Laminaria in 1914 by Willstätter and Page [27]. Since 2017, an increasing number of research publications on fucoxanthin with pharmacological properties have been observed (7). Thereafter, the number of yearly publications has dramatically increased to date. However, it slightly decreased in 2020 (14) (Figure 2A), indicating the increased attention of the scientific community to the health-promoting potentials of fucoxanthin. In addition, the last five years (2017–2021) of publication on overall fucoxanthin was recorded as 35% (P 2021). Of these, the highest number of documents has been in China (38%), followed by South Korea (14%), Japan (8%), and so on (Figure 2B). Of the total literature published on fucoxanthin, research articles made up the lion’s share, accounting for 67% (Figure 2C), while the review article shared a quarter of the total (27%) (Figure 2C), which further indicated its enormous popularity in the scientific community. Moreover, some document types are reported to be very low in number, corresponding to zero as a percentage. Considering the recent reports on the total pharmacological potentials of fucoxanthin, anticancer and antitumor research remained at the top (16%), followed by anti-inflammatory (15%), antioxidant (11%), neuroprotective (10%), and anti-hyper-lipidemia and anti-obesity (9%). The protective effects of fucoxanthin in hepatic, diabetic, kidney, cardiac, skin, and respiratory diseases were almost equally significant (4~5%). With respect to the model system utilized to assess the pharmacological properties of fucoxanthin, in vivo studies were conducted in the highest number (60), followed by human cells (32) and animal cells (33). In the last five years, only one clinical study was reported on the protective effects of fucoxanthin against obesity [15]. Despite the large number of health benefits of fucoxanthin, as marked by in vitro and in vivo evidence reviewed in the present study, the success of translational research in the clinical study is negligible. Due to its diverse health effects, the activity-wise clinical trial can be undertaken to validate the current findings using human subjects before being developed as a pharmaceutical drug of a single compound with multi-organ targets.

### 2.2. Structural Characteristics of Fucoxanthin

Fucoxanthin is a naturally occurring marine carotenoid, also known as marine xanthophyll, and is abundantly found as a pigment in the chloroplasts of brown algae. The structure of fucoxanthin is closely related to peridinin, neoxanthin, and dinoxanthin. Similar to other carotenoids, fucoxanthin obtains unique characteristic features of an unusual allenic bond, a 5,6-monoepoxide, nine conjugated double bonds, and oxygenic functional groups [2]. Moreover, the oxygenic functional groups consisting of epoxy, hydroxyl, carboxyl, and carbonyl moieties exceptionally express the superiority of fucoxanthin over other carotenoids (Figure 1). Moreover, the chemical formula of fucoxanthin is C_42_H_58_O_6_, which corresponds to a molecular weight of 658.9 g/mol. Fucoxanthin is readily vulnerable to any of the following factors, including light, heat, oxygen, enzymes, unsaturated lipids, and prooxidant molecules [2]. Depending on the treatment conditions, carrier molecules and carotenoid types may eventually cause cis-isomers formation by isomerization. Fucoxanthin extracted and purified from microalgae yielded three distinct peaks in which there are one trans-form and two cis-isomers in the chromatogram [2]. The trans-form of fucoxanthin is closely related to fucoxanthin’s pharmacological activities, as evidenced by previous studies. In a study conducted by Kawee-ai et al. [28], the antioxidant activities were decreased with the increase of cis-isomers formation. The spectrophotometric analysis of fucoxanthin-rich canola oil was subjected to different temperature exposures (between 25 and 100 °C) without light and air, resulting in the degradation of all-trans isomers of fucoxanthin, rather increasing 13-cis and 13′-cis isomers. This process followed simple first-order kinetics. Moreover, a similar pattern of degradation of fucoxanthin was observed when exposed to light and air. All studies shed light on the background knowledge of the structural properties of fucoxanthin, with the intent of taking preventive measures against chemical degradation when used for pharmaceutical purposes.

### 2.3. Pharmacological Properties of Fucoxanthin Evidence from In Vitro and In Vivo Studies

#### 2.3.1. Antioxidant Activity

Oxygen has been the inevitable molecule for all living cells. Molecular oxygen plays a pivotal role in the regulation of cellular maintenance and energy production with the concomitant generation of ROS. Excess levels of ROS generate various pathological events, along with poor antioxidant defense systems in living organisms. Antioxidants are likely to be scavenged free radicals, viz. singlet oxygen, hydrogen peroxide, superoxide anion, and DPPH to rescue cells from the devastating effects of oxidative stress (33, 34). Oxidative stress is the consequence of an imbalanced situation between oxidant and antioxidant molecules [29]. Marine carotenoid fucoxanthin has been reported as the most promising antioxidant metabolite with potent resistance against ROS and/or oxidative stress in vitro and in vivo (Table 1).

An in vitro cell-free assay resulted in antioxidant activities of fucoxanthin extracted from brown algae, with an increased level of DPPH radical scavenging activity and iron-chelating activity and decreased reducing power in the in vitro cell-free assay [30], and later, similar DPPH activity was found by Mousavi et al. [31]. Fucoxanthin isolated from *Phaeodactylum tricornutum* showed promising antioxidant activities when added to different cell lines including RAW 264.7, HepG2, Caco-2, and HeLa cells; these results were caused by decreased metabolic activity and chemiluminescence and an increased antioxidant glutathione level [7]. In studies with an animal model, the administration of fucoxanthin to ovalbumin-induced asthma mice resulted in decreased intracellular ROS formation and increased antioxidant enzyme activity [32], and in alcoholic liver injury mice yielded elevated levels of liver total antioxidant capacity (T-AOC), glutathione peroxidase (GSH-Px), superoxide dismutase (SOD), and catalase (CAT), probably through the activation of the Nrf2-mediated antioxidant pathway [9]. A similar Nrf2-mediated antioxidant pathway was further characterized by Chen et al. [33] when fucoxanthin was administered in LPS-induced uveitis rats, in addition to increased SOD and decreased malondialdehyde (MDA). Chiang and the team experimented on diabetic retinopathy in human retinal epithelial cells that showed protective effects of fucoxanthin to increase the antioxidant enzyme activity and decrease ROS levels, probably through the strong antioxidant activities of Nrf2 activation [23]. When fucoxanthin was added to sunscreen (0.5% *w*/*v*), it provided a significant inhibition of ROS formation in reconstructed human skin [34]. In TGFβ1-induced oxidative stress with excessive ROS accumulation in the human hepatic stellate cells model, primary metabolites of fucoxanthin such as fucoxanthinol and amarouciaxanthin A were treated in cells that showed preventive effects by antioxidant potentials by a reduced level of ROS and the increased expression of Nrf2 proteins [6]. In another study conducted by Yang and the team [8], the peroxidase, SOD, CAT, and ascorbate peroxidase in cadmium-exposed thyroid tissues in mice were decreased, and however, significantly recovered after fucoxanthin administration, through antioxidant activities together with downregulated ERK-mediated apoptosis pathways. Accumulating results suggest that fucoxanthin as an antioxidant with a multitude of protective functions in the in vitro and in vivo studies can be a lead compound for future translational research into clinical trials. Additionally, further research activities can be undertaken to promote fucoxanthin as a nutraceutical for antioxidants in food, medicine, and cosmetics.

**Table 1:** An updated summary of antioxidant activities of fucoxanthin: In vitro and in vivo studies.

Experimental Model (In Vitro/In Vivo)	Treatment (Dose, Route and Duration)	Major Outcomes	Reference
In vitro cell-free assays	0.01–2 mg/mL extracted from *F. vesiculosus*, *F. serratus*, and *L. digitata* in 5% fish oil in water emulsion; butylated hydroxytoluene as positive control	↑ DPPH scavenging and iron-chelating activity; ↓ reducing power	[30]
LPS-induced RAW 264.7 and HepG2, Caco-2 and HeLa cells	0.1–50 μg/mL (purity ≥ 99.2%) extracted from *P. tricornutum* in 0.1% DMSO, pre-treatment for 24 h; staurosporine 1 μM as positive control	↑ DPPH activity with IC50 value of 201.2 ± 21.4 µg/mL ↓ metabolic activity and caspase 3/7	[7]
OVA-induced-asthma mouse	50 mg/kg b.w., treatment (N/A)	↓ ROS; ↑ antioxidant enzyme activity; ↓ inflammatory cytokine markers	[32]
Alcohol-induced liver injury in mice	10–40 mg/kg, orally for 7 days; silibinin 80 mg/kg as positive control	↑ T-AOC, GSH-Px, SOD and CAT; ↑ Nrf2, NQO1, HO-1 and GCLM	[9]
4-HNE induced-diabetic retinopathy in ARPE-19 cells	0.1–0.5 mg/mL, post-treatment for 24 or 72 h	↑ Cell viability; ↓ DNA damage; ↓ cleaved PARP; Nrf2 protein; ↓ ICAM-1 protein expression; ↑ ZO-1 expression; ↓ ROS; ↑ CAT	[23]
In vitro cell-free assays	0.05–0.3 mg/mL extracted from Isochrysis galbana	↑ DPPH activity with EC50 value of 0.2 mg/mL	[31]
UVA-induced reconstructed human skin tissue	0.5% extracted from *D. anceps*, pre-treatment for 1 h	↓ intracellular ROS	[34]
LPS-induced uveitis in rats	1–10 mg/kg b.w. in 0.1% DMSO, Orally for 7 days	↑ Nrf2 in ocular tissues; ↑ SOD; ↓ MDA	[33]
TGFβ1-induced fibrosis in human LX-2 cells	FxOH 0.1–0.5 μM (purity ≥ 97%) and AcxA 0.2–1 μM (purity ≥ 97%) in DMSO, pre-treatment for 1–24 h	↓ ROS; ↑ Nrf2 expression	[6]
Cadmium-induced thyroid gland injury mice	10–50 mg/kg b.w., orally for 14 days; thyroid tablets 50 mg/kg as positive control	↑ POD, SOD, CAT and APX; ↓ mRNA expressions of ERK1 and 2, caspase3, 8 and 9	[8]

↑: upregulation; ↓: downregulation.

#### 2.3.2. Anti-Inflammatory Activity

Inflammation is an essential immune response to restore the body’s unstable physiological homeostasis due to various stresses, infections, injuries, etc. Despite its protective role, such responses are expected to persist for a short time without interfering with regular cellular functions. The excessive production of characteristic proinflammatory cytokines (TNF-α, IL-1β, IFN-γ, IL6 and CCL5), mediators (PGE2, LT), nitric oxide, free radicals, etc. has made inflammation a complex process [11]. With the in vitro and in vivo studies, scientists continue their research endeavor to understand underlying molecular mechanisms of inflammation with the aim of discovering effective anti-inflammatory therapies from natural sources such as fucoxanthin (Table 2).

Fucoxanthin treatment in LPS-generated inflammation in RAW 264.7 macrophage resulted in the lower expression of Tnfα, Il1b, and Il6 mRNA and TNFα, due to abated Nrf2 nuclear translocation via PI3K/AKT signaling pathway [35]. Li and co-investigators reported the anti-inflammatory effects of fucoxanthin on the palmitate-activated 264.7 macrophage [36]. It revealed the attenuation of IL-6, IL-1β, TNF-α, and NLRP3 inflammasome genes and the increment of pAMPK, indicating the anti-inflammatory contribution of fucoxanthin. It also promoted lipid metabolism through an increase of CPT1a, PPAR γ to prevent free fatty acid-induced inflammation. Fucoxanthin administration to hepatocyte injury mice caused by nonalcoholic steatohepetitis (NASH) [37] and treatment of LPS-induced RAW 264.7 macrophages cells [38] significantly decreased the expression of inflammatory genes such as IL-1β, IL-6, IL-8, and TNF-α, respectively. Similar inflammatory gene expression was observed in UVA-induced reconstructed human skin [39]. In cultured human keratinocytes, fucoxanthin reduced COX-2, PGE2, ILO-6, IL-1β, and TNF-α, iNOS, and PGE2 levels in particulate matter (PM)-induced macrophages [40]. Moreover, in PM-induced zebrafish embryos, fucoxanthin attenuated NO and ROS levels and thereby inflammation. Zhao and the team showed the anti-neuroinflammatory activity of fucoxanthin on LPS-induced BV-2 microglia. The study demonstrated the reduced secretion of pro-inflammatory mediators such as TNF-α and IL-6, and the mRNA expression of COX-2 and iNOS through the downregulation of Akt/NF-κB and MAPKs/AP-1 pathways [11]. In addition, fucoxanthin provided protection against neurodegenerative diseases by both enhancing brain-derived neurotrophic factor (BDNF) and subsiding pro-inflammatory mediators. In the LPS-induced mouse model, fucoxanthin treatment demonstrated its role as an antidepressant in MDD (major depressive disorder) via the regulation of the AMPK/NF-κB pathway to attenuate the secretion of neuroinflammatory factors (Cox-2 and iNOS; and pro-inflammatory mediators (TNF-α, IL-6 and IL-1β)) in the hypothalamus, frontal cortex, and hippocampus [41]. Chen and colleagues showed the preventive role of fucoxanthin on the inflammation in the UV-B-induced eye of rats. It enhanced Nrf2 following the downregulation of p38 MAPK, GFAP, and TRPV1 [42]. In the ulcerated colon from exposure to dextran sulfate sodium (DSS), fucoxanthin downregulated the NF-κB pathway and PGE2 production [10].

In the case of HFD-induced hepatic inflammation (non-alcoholic steatohepatitis, NASH) in mice, fucoxanthin diet decreased the mRNA expression of inflammatory cytokines (IL-1β, IL-6, TNFα) and chemokine (MCP1). Fucoxanthin also extenuated MCP-1, CCL5, IL-8I, and Th2 (L-4, IL-5 and IL-13) expressions in ovalbumin-activated asthmatic mice [43]. Apart from brown macroalgae, fucoxanthin extracted from diatoms was also found to inhibit the NF-κB pathway in both the LPS-activated septic mouse model and macrophage 264.7 cells [44]. Lee and co-workers have revealed that fucoxanthin from microalga, Phaeodactylum tricornutum, caused the downregulation of NF-κB signaling and initial blockage to NLRP3 inflammasome by attenuating caspase1 and apoptosis-associated speck-like protein containing a CARD (ASC), respectively, in LPS or, LPS/ATP induced bone marrow-derived macrophages (BMDMs), bone marrow-derived dendritic cells (BMDCs), and in astrocytes [45]. This mounting evidence points to the therapeutic potential of fucoxanthin and its metabolites in healing inflammatory situations. However, further clinical trials may explain the underlying mechanisms of fucoxanthin’s anti-inflammatory activity, elucidating any possibilities of its collateral damages.

**Table 2:** An updated summary of anti-inflammatory activities of fucoxanthin: In vitro and in vivo studies.

Experimental Model (In Vitro/In Vivo)	Treatment (Dose, Route, and Duration)	Major Outcomes	Reference
LPS-activated BV-2 microglia	5–20 μM (purity ≥ 98%), pre-treatment for 1 h	↓ IL-6, TNF-α, PGE2, NO, iNOS, COX-2 enzymes; ↑ Nrf-2 activation; ↑ HO-1 expression; ↑ BDNF; ↓ Akt/NF-κB; ↓ MAPKs/AP-1	[11]
UV-B-stimulated corneal denervation in rats	0.1 to 10 mg/kg b.w., orally for 6 days	↑ Nrf2 in cornea; ↓ p38 MAPK; ↓ GFAP-positive neural cells; ↓ TRPV1 expression in the trigeminal ganglia neurons	[42]
LPS-treated mice	50–200 mg/kg b.w. in 0.5% sodium carboxymethylcellulose, intragastric route for 7 days	↑ AMPK; ↓ NF-κB; ↓ TNF-α, IL-1β, IL-6; ↓ iNOS and COX-2	[41]
LPS-induced sepsis mouse model	0.1–10 mg/kg b.w. extracted from *Conticribra weissflogii* ND-8, intraperitoneally for 6–120 h; ulinastatin as positive control	↓ IL-6, IL-1β and TNF-α;	[44]
LPS-induced RAW 264.7 cells	10 nM extracted from *Conticribra weissflogii* ND-8, co-treatment for 6 h	↓ NF-κB signaling pathway	
Palmitate-activated RAW 264.7 cells	50 μM (purity ≥ 95%), co-treatment for 12 h	↓ IL-6, IL-1β, TNF-α and NLRP3 gene; ↑ *TGF β* gene; ↑ CPT1a, PPAR γ; ↑ pAMPK	[36]
CDAHFD-induced NASH model mice	0.2%/day extracted from brown seaweed lipid, orally for 4 weeks	↓ Hepatic IL-1β, IL-6, TNF-α mRNA expression; ↓ MCP-1 mRNA expression; ↓ serum MCP-1	[37]
UVA-induced reconstructed human skin	all-trans fucoxanthin (0.5% *w*/*v*) (purity ≥ 95%) in Alkyl benzoate and ethanol, co-treatment for 15 min; sodium dodecyl sulfate as positive control	↓ IL-6, IL-8 gene expression	[39]
DSS-stimulated ulcerative colitis mice	50–100 mg/kg b.w., treatment (NA)	↓ PGE2, COX-2; ↓ NF-κB	[10]
LPS-induced RAW 264.7 macrophages	4.7–470 ng/mL (purity ≥ 95%) extracted from T. lutea F&M-M36, co-treatment for 18 h; celecoxib 3 μM as positive control	↓ IL-6; ↑ IL-10, Arg1	[38]
PM-induced zebrafish embryo	25–100 μg/mL extracted from Sargassum fusiformis, co-treatment for 72 h	↓ NO, ROS	[40]
PM-activated HaCaT keratinocytes and RAW 264.7 cells	25–100 μg/mL extracted from Sargassum fusiformis, co-treatment for 30 min	↓ NO, IL-1β, TNF-α and IL-6; ↓ PGE2, COX-2 and MAPK	
LPS-activated RAW 264.7 cells	5 μM (purity ≥ 95%), pre-treatment for 12 h	↓ IL6, IL-1β and TNF mRNA; ↓ TNFα secretion; ↓ PI3K/AKT/ Nrf2	[35]
LPS/ATP-stimulated BMDMs and BMDCs	40 μM extracted from Phaeodactylum tricornutum, pre-treatment for 4 h	↓ IL-1β, IL-6 and TNF-α; ↓ NLRP3, ASC and cleaved caspase-1; ↓ oligomerization of ASC; ↓ NF-κB	[45]
LPS-induced RAW264.7 cells	2.5 μM (purity ≥ 96%) fucoxanthinol from fucoxanthin, extracted from brown seaweed lipid, co-treatment for 24 h	↓ proinflammatory mediators; ↓ MAPK/NF-κB signaling pathways	[46]
OVA-triggered asthmatic mice	10–30 mg/kg b.w. (purity ≥ 95%) in DMSO, intraperitoneally for 28 days; prednisolone 5 mg/kg as positive control	↓ IL-8, MCP-1 and CCL5; ↓IL-4, IL-5, IL-13; ↑ IFN-γ expression	[43]

↑: upregulation; ↓: downregulation.

#### 2.3.3. Anticancer and Anti-Tumor Activity

Cancer, one of the leading causes of death, hinders the attainment of a sufficient life expectancy in every corner of the globe. An estimated 19.3 million new cancer cases and almost 10.0 million cancer deaths were reported in 2020 [47]. Now, the underlying molecular pathways are unveiled, paving the way for introducing a plethora of anticancer drugs, thanks to the state-of-the-art technology and research. However, synthetic drugs have failed to improve overall patient survival, driving the scientific community to develop novel therapeutic agents. Phytochemicals could be a potential source of novel anticancer drugs. The potential of fucoxanthin has also been explored as an anticancer agent against various cancer types (Table 3). The cell tumorgenicity and sphere formation observed in human colorectal cancer cells and mice with tumors were attenuated when fucoxanthinol, a metabolite of fucoxanthin, was added to the culture, via the downregulation of pAkt, peroxisome proliferator-activated receptor (PPAR)p/8 and PPARγ with increased chromatin condensation and nuclear fragmentations [48]. Another study by Terasaki et al. [14] found that the oral administration of fucoxanthin in azoxymethane/dextran sodium sulfate (AOM/DSS)-induced colorectal cancer mice reduced the incidence and the multiplicity of colonic adenocarcinoma, by downregulating the integrin signaling pathway. Later, in 2021 and 2022, Terasaki and the team performed anti-tumor activities in the mice model after exposure to (AOM/DSS), in which fucoxanthin administration resulted in the suppression of the number of colorectal adenocarcinomas and cyclin D1 levels [49], and the signals including Ccr1, Cyclin D1, pSmad2, MAPK, PI3K/AKT, p53, RAS, STAT, TGF-β, and Wnt by transcriptome analysis [50].

Almeida and co-workers [13] showed that fucoxanthin possesses an anti-proliferative effect on two human leukemia cell lines, K562 and TK6. Fucoxanthin also displayed anticancer activity on the breast cancer cell line and human glioblastoma cell line via decreasing cell viability, proliferation and clonogenic potential, migration and invasion, tubulogenesis, and angiogenesis [51,52]. Fucoxanthin effectively induced apoptosis and cell-cycle arrest in gastric adenocarcinoma SGC-7901 and BGC-823 cells via down-regulating the expression of Mcl-1, STAT3, and p-STAT3, and the regulation of the JAK/STAT signal pathway [53]. Fucoxanthin treatment caused the cytotoxicity and death of HepG2 cancer cells via apoptotic, antioxidant, and anti-inflammatory pathways [54,55]. By decreasing VEGF-C, VEGF receptor-3, nuclear factor kappa B, phospho-Akt and phospho-PI3K, micro-LVD, fucoxanthin showed anticancer activity in human breast cancer MDA-MB-231 cells [12].

Fucoxanthin also effectively suppressed the cell proliferation and colony formation in cervical cancer cells in molecular mechanisms of inducing apoptosis and decreasing the expression of histone cluster 1 H3 family member D (HIST1H3D) [56]. Murase et al. [57] reported the anti-tumor effects of fucoxanthin in c57bl/6j mice with decreased expressions of the chemokine (C-C motif) ligand 21 (CCL21)/chemokine receptor 7 (CCR7) axis. Fucoxanthin causes epigenetic and transcriptomic reprogramming, which potentially contributes to the protection of TPA-induced JB6 cellular transformation skin cancer in an animal model [58]. Some reports also suggest that fucoxanthin can be used in combination with other chemotherapy agents to achieve the maximum benefit of chemotherapy [59,60]. The above preclinical evidence suggests that fucoxanthin might have the potential to curb one of the challenging diseases of our time to be considered as a novel therapeutic agent against cancer.

**Table 3:** An updated summary of anti-cancer and anti-tumor potentials of fucoxanthin: In vitro and in vivo studies.

Experimental Model (In Vitro/In Vivo)	Treatment (Dose, Route and Duration)	Major Outcomes	Reference
CCSCs, CD44high/EpCAMhigh tumor cells and HT-29 human colorectal cancer cells	0.1–5.0 µM fucoxanthinol from fucoxanthin (purity ≥ 98%) in tetrahydrofuran, treatment for 5 days	↓ Cells viabilities; ↓ pAkt, PPARβ/δ and PPARγ; ↓ Colonospheres growth; ↑ Chromatin condensation; ↑ Nuclear fragmentations	[48]
NOD-SCID mice with tumors	5 mg/kg b.w. fucoxanthinol from fucoxanthin (purity ≥ 98%) in tetrahydrofuran, orally every 3–4 days for 2 weeks	↓ Csps tumorigenesis	
Leukemia cell lines, K562 and TK6	0.1–10 μM in DMSO, treatment for 24 h	↓ Cell viability and proliferation; ↓ Nuclei size; ↓ Anti-apoptotic protein (bcl-2 and caspase-3)	[13]
Breast cancer cells line, MDA-MB-231 and normal human skin fibroblast cells line	10–50 μg/mL extracted from *P. tenuis*, *C. sinuosa*, *I. stellate* and *D. indica* in DMSO, treatment for 6–48 h	↑ Death of cancer cells; ↓ Cell viability	[51]
Human gastric adenocarcinoma SGC-7901 or BGC-823 cells	25–75 μM (purity ≥ 99%) extracted from Undaria pinnatifda in ethanol, pre-treatment for 24 h; paclitaxel 1 μM as positive control	↑ Apoptotic cells; ↓ Cells cycle at S phase (SGC-7901) and G2/M phase (BGC-823); ↓ Mcl-1, STAT3 and p-STAT3	[53]
Benzo(A)pyrene-induced lung cancer mice	N/A	↑ Apoptosis (Caspase 9 and 3); ↓ Anti-apoptotic protein (Bcl2); ↓ Expression of PCNA	[54]
Human liver HepG2 cancer cell line	10-40 μgmL^−1^ extracted from Chaetoceros calcitrans in DMSO, treatment for 72 h; doxorubicin as positive control	↓ Proliferation; ↓ AKT1, ERK ½, JNK expression; ↑ BAX and BID gene; ↑ APAF and CYCS expression; ↓ Antioxidant genes (SOD1, SOD2, CAT)	[55]
Human breast cancer MDA-MB-231 cells	25–100 μM extracted from U pinnatifida, treatment for 12–48 h	↓ Lymphangiogenesis; ↓ VEGF-C, VEGF receptor-3, NF-κB, p-Akt and p-PI3K, micro-LVD	[12]
GBM1, A172 and C6 cell lines	10–150 μM extracted from Phaeodactylum tricornutum, treatment for 24 h	↓ Cell viability and proliferation and invasion; ↓ Angiogenesis and tubulogenesis; ↓ ATP levels; ↑ Apoptosis	[52]
AOM/DSS-induced carcinogenic mice	30 mg/kg b.w. in palm oil, orally every 1 or 3 days for 3 weeks	↑ Mucosal crypts and anoikis-like integrin 1^low/-^/cleaved caspase-3^high^ cells; ↓ Integrin阝, pFAK, pPaxillin, αSMA	[14]
HeLa and SiHa cervical cancer cells	0.1–25 µM, treatment for 48 h	↓ Hela and SiHa cells (IC50: 1445 and 1641 µM, respectively) ↑ apoptosis; ↓ cell proliferation and colony formation; ↓ HIST1H3D and its mRNA, cell cycle at G0/G1 phase	[56]
Human non-small cell lung cancer A549, H1299, PC9 and small cell lung cancer H446 cell lines	5–30 μM (purity ≥ 99%) extracted from Laminaria Japonica in ethanol, treatment for 48 h; diamminedichloroplatinum 5 mg/kg as positive control	↓ Cells migration and invasion, metastasis; ↓ Expressions of Snail, Twist, Fibronectin, N-cadherin, MMP-2, PI3K, p-AKT and NF-Κb; ↑ Expression of TIMP-2	[60]
C57BL/6J mice, orthotopic transplantations of cancer cells (KMPC44)	3 mg/g b.w. in palm oil, orally for 2 weeks	↓ Adenocarcinoma; ↓ CCL21/ CCR7 axis, Rho A, BTLA, N-cadherin, SMA, pFAK and pPaxillin	[57]
AOM/DSS-induced colorectal tumorigenesis in ApcMin/+ mice	30 mg/kg b.w., orally for 5 weeks	↑ Cleaved caspase-3; ↓ cyclin D1 expression; ↓ Bacteroidlales and Rikenellaceae; ↑ Lachnospiraceae	[49]
AOM/DSS-induced colorectal cancer mice	50 mg/kg b.w., orally for 14 weeks	↓ Ccr1, Cyclin D1, pSmad2, MAPK, PI3K/AKT, p53, RAS, STAT, TGF-β and Wnt	[50]
TPA-induced skin cell transformation in Nfe2l2wild-type cells	N/A	↓ ROS, oxidized GSSG/reduced GSH	[58]

↑: upregulation; ↓: downregulation.

#### 2.3.4. Anti-Hyperlipidemic and Anti-Obesity Potentials

Obesity, especially the central one, has a long history of having association with hyperlipidemia, for example, increased plasma triglycerides, high LDL cholesterol, low HDL cholesterol, etc., are responsible for higher blood glucose, insulin levels and high blood pressure, and all are related to increased cardiovascular risk. Empirical evidence indicates that fucoxanthin can possess anti-obesity and anti-hyperlipidemic potentials (Table 4). In a double-blind placebo-controlled study conducted by Hitoe et al. [15], capsules containing 1 and 3 mg fucoxanthin were administered to mildly obese males and females for 4 weeks, resulting in reduced body weight, BMI, and fat area and mass. Fucoxanthin-rich phaeodactylum tricornutum or Padina tetrastromatica extract ameliorated the effects of high diet-induced obesity in c57bl/6j mice by inhibiting adipocytic lipogenesis, inducing fat mass reduction, and decreasing intracellular lipid content, adipocyte size, and adipose weight [61,62,63]. Interestingly, fucoxanthin has modulatory effects on gut microbiota to provide anti-obesity effects [64]. In addition, fucoxanthin alleviated HFD-induced gut microbiota dysbiosis by significantly inhibiting the growth of obesity-/inflammation-related Lachnospiraceae and Erysipelotrichaceae while promoting the growth of Lactobacillus/Lactococcus, Bifidobacterium, and some butyrate-producing bacteria [17]. Similarly, the dietary supplementation of fucoxanthin in HFD-induced obese mice improved obesity, hyperglycemia and hyperlipidemia, and alleviated insulin resistance, possibly through the regulation of IRS-1/PI3 K/Akt and PPARγ/SREBP-1/FAS signaling pathways [65]. Meanwhile, through IRS-1/PI3K/Akt and AMPK pathways, fucoxanthin ameliorates hyperglycemia, hyperlipidemia, and insulin resistance in diabetic mice [66]. In oleic acid-induced fatty liver cells, fucoxanthin significantly suppressed lipid accumulation and decreased lipid peroxidation in hepatocytes through the Sirt1/AMPK pathway [16].

#### 2.3.5. Antidiabetic Activity

Diabetes mellitus (DM) is a chronic metabolic illness marked by hyperglycemia, disturbance in glucose, protein, and fat metabolism, leading to disruptions in insulin secretion, action, or both. Although type-1 diabetes causes insulin deficiency by the autoimmune-mediated destruction of pancreatic β-cells, type-2, on the other hand, causes peripheral insulin resistance as a result of either defective insulin secretion by pancreatic β-cells or an inability of insulin-sensitive tissues to respond appropriately to insulin. Moreover, both varieties (type-1 and type-2) can cause complications such as diabetes neuro, nephro, and retinopathy (DR), and ultimately lead to dysfunction in multiple body parts. The increasing number of pieces of evidence on the use of marine bioactive substances such as fucoxanthin in treating diseases like diabetes has proven the reliability of medicinal remedies compared to the side effects of existing drugs in cellular and animal studies (Table 5). For example, fucoxanthin, after high glucose and lipid peroxidation stress-induced DR, protected against inflammatory responses, maintained the integrity of the blood–retinal barrier by reducing cell damage, i.e., by reducing apoptosis and cell adhesion factor protein expression [23]. Meanwhile, in streptozotocin-induced diabetic rats, fucoxanthin effectively improved renal fibrosis by activating Sirt1/Nrf2 signaling to reduce oxidative stress [67]. In addition, a dietary fucoxanthin supplement in C57BL/KsJ-db/db mice at 0.2% and 0.4%, *w*/*w* for 6 weeks significantly improved glucose/lipid metabolism and insulin resistance, and prevented pancreatic histological changes by the effective regulation of the expression of IRS-1/PI3K/AKT and AMPK signaling proteins in liver and skeletal muscle [66]. Likewise, fucoxanthin from brown algae improved male reproductive function, not only by possessing antioxidant and anti-inflammatory properties, but also decreased the diabetic signs in streptozotocin/nicotinamide-induced diabetic rats [3,68].

#### 2.3.6. Cardioprotective Activity

Cardiovascular diseases (CVDs) are the most common cause of death worldwide, killing around 17.9 million people each year [69]. Some CVDs involve inflammatory reactions leading to oxidative damage and apoptosis [70]. The acute myocardial infarction is also associated with elevated levels of ROS and oxidative stress [71]. In different studies, fucoxanthin showed cardioprotective effects by attenuating oxidative stress and apoptosis (Table 6).

Fucoxanthin improved the ventricular rhythm and muscular function in the aging C57BL mice model. The study was performed with both low molecular weight fucoidan (LMWF) and high stability fucoxanthin, alone and in combination. Fucoidan and fucoxanthin improved the cardiac morphology, and ameliorated the expression of the son of sevenless 1 (SOS1), growth factor receptor-bound protein (GRB2), glycogen synthase kinase 3 beta (GSK3β), cAMP-response element binding protein (CREB), and insulin receptor substrate 1(IRS) [72]. In another study of the doxorubicin-injected ICR mice model, fucoxanthin prevented cardiotoxicity. It significantly reduced AST, LDH, and creatine kinase-MB (CKMB). In the in vitro study of rat cardiomyocytes, fucoxanthin prevented doxorubicin-induced oxidative damage and apoptosis. The antioxidative and anti-apoptotic effects of fucoxanthin involve p38, c-Jun N-terminal kinases (JNK) and p53 pathways [21]. Fucoxanthin also exhibited cardioprotective activity in isoprenaline hydrochloride-induced myocardial rats. It reduced troponin T, troponin I, and thiobarbituric acid reactive substances (TBARS). In addition, it promoted antioxidant activity by elevating SOD, CAT, and GPx; and reduced inflammation by downregulating NF-κB, IL-6, and TNF- α [73]. In another study, H_2_O_2_ induced oxidative stress and subsequent apoptosis in rat valve interstitial cells. Fucoxanthin prevented oxidative stress-induced apoptosis by enhancing antioxidant activity and downregulating apoptotic markers caspase-3, caspase-8, and caspase-9, etc. Furthermore, it aided cell survival and protected from calcification [70].

#### 2.3.7. Hepatoprotective Activity

Hepatic disease causes about 2 million deaths worldwide annually [74]. Moreover, the modern lifestyle is much more sedentary, and may increase the burden of hepatic disease in the coming days, so there is an urgent need to find novel therapeutic approaches to address this global issue. As a prospective hepatoprotective agent, fucoxanthin potential has been explored in cellular and animal models. Some of the studies investigating the hepatoprotective effects of fucoxanthin and the underlying molecular mechanisms are summarized in this review (Table 7). For example, fucoxanthin reduced fatty inclusion in FL83B hepatocytes via modulating the Sirt1/AMPK signaling pathway [16]. Fucoxanthin conferred AMPK-mediated cytoprotection and promotes autophagy in HepG2 cells under oxidative stress via the upregulation of autophagic markers (LC3II and beclin-1), the activation of AMPK, and the downregulation of p-mTOR [75]. The hepatocarcinogenesis of rat was used to examine the protective effects of fucoxanthin, which increased serum SOD, CAT, and GPx, and decreased alanine aminotransferase (ALT), AST (aminotransferase), alkaline phosphatase (ALP), lactate dehydrogenase (LDH), gamma-glutamyl transferase (GGT), and serum bilirubin and stress markers [76]. In addition, fucoxanthin exhibited protection against alcoholic liver injury via the activation of Nrf2-mediated antioxidant defense and the inhibition of TLR4-mediated inflammation [9].

#### 2.3.8. Reno-Protective Activity

Kidney disease is a major public health concern around the world. Inflammation, oxidative stress, apoptosis, and fibrosis are the leading causes of the development of acute kidney injury (AKI) and chronic kidney disease (CKD) [77]. Evidence suggests that patients with a history of AKI are more likely to develop CKD [78]. The progression of CKD increases the chance of death and leads to end-stage renal disease. Fucoxanthin exhibited reno-protective effects in several studies, as summarized in Table 8. Fucoxanthin affected diabetic nephropathy by alleviating oxidative stress and fibrosis. In the high glucose (HG)-treated mesangial cells, fucoxanthin significantly reversed the HG-induced expression of fibronectin (FN), collagen IV, and reactive oxygen species (ROS). A reduction in oxidative stress and fibrosis by fucoxanthin involves the Akt/Sirt1/FoxO3α signaling pathway [79]. In another study of ethylene glycol-treated albino rats, fucoxanthin normalized the elevated renal stone and biochemical markers. Fucoxanthin treatment also significantly reduced oxidative stress and lipid peroxidation. In addition, it prevented calculi deposition and tubular damage [80]. Fucoxanthin treatment attenuated mesangial cell damage in the HG-induced diabetic nephropathy (DN) disease model [20]. Moreover, it significantly reduced HG-elevated ROS levels and enhanced antioxidant activity [20,67]. In the same study, in the streptozotocin (STZ)-induced diabetic rat model, fucoxanthin improved renal function and reduced fibrosis [67]. In the cadmium chloride-treated mice model, fucoxanthin protected the kidney by inhibiting oxidative stress and apoptosis, and recovering mitochondrial structural integrity. It significantly reduced apoptosis-related markers such as caspase-3 and caspase-9; and improved antioxidant activity by increasing the levels of SOD, CAT, and peroxidase (POD) [8].

#### 2.3.9. Ocular Protective Activity

Ultraviolet (UV) radiation can cause serious injury to the eyes after exposure, resulting in an opaque cornea and decreased visual acuity, due to severe oxidative stress that triggers ROS production and inflammatory cytokines. It has been evidenced by many studies on animal and human subjects [81]. To establish the ocular protective functions of fucoxanthin, as shown in Table 9, UVB-induced corneal denervation and trigeminal pain in the rat model were performed, where fucoxanthin increased Nrf2 expression in the cornea and decreased p38 MAP kinase, GFAP-positive neural cells, TRPV1 expression in the trigeminal ganglia neurons, and eye-opening and wipe behavior [42]. In another study investigated by Chiang et al. [23], diabetic retinopathy induced by Glucose- and 4-Hydroxynonenal (HNE) in human retinal pigment epithelial cells, fucoxanthin showed protective effects by reversing cell damage, inflammatory response, cell adhesion factor protein expression, and apoptosis through the modulation of Nrf2 signaling pathways. The lipopolysaccharide-induced uveitis rats model has been reported to have similar ocular protective Nrf2 signaling pathways by fucoxanthin, as characterized by increased SOD level, and decreased MDA level, inflammatory cells, TNF-α concentrations, and corneal endothelial disruption [33]. Accumulating scientific evidence suggests that fucoxanthin is a potent dietary carotenoid with antioxidant and anti-inflammatory potentials to protect eyes against injuries.

#### 2.3.10. Neuroprotective Activity

The brain is consistently exposed to numerous toxic insults that lead to oxidative stress and neuroinflammation, causing pathological changes in brain tissues [82]. These phenomena are thought to be implicated in the pathobiology of neurodegenerative disorders (such as AD and PD) and secondary damage from brain injury (such as ischemic stroke and traumatic brain injury) that contribute to the major causes of cognitive deficits among the elderly. Numerous bioactive compounds, including marine natural products, were shown to be effective in attenuating neuronal injury and improving cognitive deficits [83].

A growing body of evidence from cellular and animal studies has claimed the promising neuroprotective effects of fucoxanthin (Table 10). A study shows that fucoxanthin inhibited β-amyloid oligomer [84] and H_2_O_2_ [85]-induced apoptosis and oxidative stress in SH-SY5Y cells through a mechanism that involves the activation of the PI3K/Akt pathway and the inhibition of the ERK pathway. In the same study by Yu and colleagues [85], fucoxanthin provided neuroprotection against H_2_O_2_-induced oxidative damage in primary cerebellar granule neurons involving a similar protective mechanism. Modified Aβ1–42 oligomers by fucoxanthin co-incubation showed less toxicity in SH-SY5Y cells compared to Aβ1-42 oligomers, suggesting that fucoxanthin mediates the structural modification of Aβ1–42 oligomers, resulting in lower neurotoxicity [18]. Another study further supports that fucoxanthin can attenuate both Aβ1-42 and H_2_O_2_-induced cytotoxicity in PC12 cells [86].

The neuroprotective effects of fucoxanthin against neurotoxic chemicals in vitro were further translated into in vivo animal models. Fucoxanthin was shown to recover cognitive deficits induced by scopolamine [18] in mice, possibly involving different mechanisms that include the inhibition of acetylcholinesterase and the attenuation of oxidative stress. Fucoxanthin ameliorated cognitive deficits, restored antioxidant and neurotransmitters levels, and reduced inflammatory markers in the rat model of streptozotocin-induced cognitive impairment [87]. In a study evaluating the brain delivery of fucoxanthin through a nanoparticle-assisted approach, poly lactic-co-glycolic acid-block-polyethylene glycol-loaded fucoxanthin (PLGA-PEG-Fuc) nanoparticles were shown to attenuate Aβ oligomers-induced neurotoxicity in neuronal and microglial cultures [88]. When administered by intravenous injection, PLGA-PEG-Fuc nanoparticles ameliorated cognitive impairments in Aβ oligomers-induced AD mice with greater potency than free fucoxanthin, possibly through involving mechanisms that include the activation of Nrf2 and NF-κB pathways [88].

In an experimental model of Parkinson’s disease, Wu and the team demonstrated that fucoxanthin protected against cellular injury in 6-OHDA-induced PC12 cells, improved the swimming capacity of 6-OHDA-exposed zebrafish larvae, and recovered the damage to the brain granular region [89]. Fucoxanthin suppresses OGD/R-induced apoptosis and ROS accumulation in cultured neurons, via activating the Nrf2/HO-1 signaling [90]. Neuroprotective effects were reported in hypoxia/reoxygenation (H/R)-induced excitotoxicity in primary hippocampal neurons when fucoxanthin and its derivative of fucoxanthinol were added to the culture [19]. Fucoxanthin also alleviated cerebral ischemic/reperfusion (I/R) injury and improved the neurologic deficit [90]. Moreover, fucoxanthin attenuates traumatic brain injury (TBI)-induced secondary damages, including neurological deficits by activating Nrf2 pathway [91]. In the TBI model of mouse primary cortical neurons, fucoxanthin prevented neuronal damage by activating the cellular antioxidant defense system [91]. The above preclinical evidence suggests that fucoxanthin could be developed as a novel therapeutic agent against degenerative brain disorders. Although the nanoparticle-guided delivery of fucoxanthin increases its bioavailability in the brain, further clinical trials are necessary to validate this claim.

**Table 10:** An updated summary of neuroprotective effects of fucoxanthin: In vitro and in vivo studies.

Experimental Model (In Vitro/In Vivo)	Treatment (Dose, Route and Duration)	Major Outcomes	Reference
β-Amyloid oligomer-induced neurotoxicity in SH-SY5Y Cells	0.3–3 μM extracted from *Sargassum horneri* (purity ≥ 90%), pre-treatment for 2 h	↓ neuronal loss and oxidative stress; ↓ ROS; ↑ pAkt and pGSK3*β*; ↓ pERK	[84]
H_2_O_2_-induced toxicity in SH-SY5Y Cells and primary cerebellar granule neurons	0.3–3 μM extracted from *Sargussum horneri* (purity ≥ 90%), pre-treatment for 2 h	↓ neuronal apoptosis and oxidative stress; ↓ ROS; ↑ pAkt and pGSK3*β*; ↓ pERK;	[85]
Aβ_1–42_ oligomers-induced neurotoxicity in SH-SY5Y Cells	0.1–1 μM extracted from *Sargussum horneri* (purity ≥ 90%), co-treatment for 24 h	↑ cell viability	[18]
Aβ oligomer-induced cognitive impairments in mice	50−200 mg/kg b.w. extracted from *Sargussum horneri* (purity ≥ 90%) in sterile saline, orally for 17 days	↑ memory formation; ↓ oxidative stress; ↑ SOD, CAT and GSH Activities; ↑ BDNF and ChAT	
Scratch-injury in cortical neurons	5–20 μM (purity ≥ 95%) in DMSO, post-treatment for 1 day	↓ MDA, GPx, ROS; ↑ viability	[91]
TBI-employed mice	50–200 mg/kg b.w. (purity ≥ 95%) in olive oil, orally for 1–7 days; 0.01–0.1 mmol/L, intracerebroventricular injection for 1–7 days	↑ Nrf2-ARE expression	
OGD/R- induced apoptosis neurons	5–20 μM (purity ≥ 95%) in DMSO, pre-treatment for 30 h	↓ Apoptosis, ROS, MDA; ↑ SOD; ↓ Cleaved caspase-3; ↑ Bcl-2/Bax expression; ↑ Nrf2 and HO-1 expression	[90]
MCAO-induced rat model (cerebral I/R injury)	30–90 mg/kg (purity ≥ 95%) in DMSO, intragastrically, 1 h before MCAO	↑ SOD activity; ↓ ROS and MDA; ↓ cleaved caspase-3; ↑ Bcl-2/Bax ratio	
H/R-induced excitotoxicity in primary hippocampal neurons	0.025–0.25 μg/mL extracted from *Undaria pinnatifida* in DMSO, co-treatment for 1.5 h of hypoxia and 24 h of reoxygenation	↑ viability; ↑ length of primary neurites	[19]
Aβ1-42- and H_2_O_2_-mediated cytotoxicity in PC12 cells	0.01–2 μM (purity ≥ 95%) in DMSO, pre-treatment for 15 min	↑ cell viability; ↓ apoptosis	[86]
Aβ oligomers-induced neurotoxicity in SH-SY5Y cells and LPS- induced neuro-inflammation in BV2 cells	PLGA-PEGFuc nanoparticles (1-10 μg/mL in 0.1% Tween-80), extracted from *Sargussum horneri* (purity ≥ 90%), co-treatment for 2 h	↑ viability; ↓ ROS; ↓ IL-1β and TNF-α	[88]
Aβ oligomers-induced recognition impairments in mice	PLGA-PEGFuc nanoparticles (i.v. 20–50 mg/kg b.w. in 0.1% Tween-80), extracted from *Sargussum horneri* (purity ≥ 90%), intravenous injection in every 2 days for 3 times	↑ cognitive performance; ↑ Nrf2; ↓ NF-κB; ↓ IL-1β and TNF-α; ↑ SOD and CAT	
Intracerebroventricular streptozotocin (ICV-STZ)-induced cognitive impairment in rats	50–100 mg/kg b.w., orally for 14 days	↑ cognitive performance; ↓ MDA and nitrite; ↑ GSH, SOD and CAT; ↓ TNF-α, IL-1β and IL-6; ↓ Aβ_(1–42)_ and Tau accumulation	[87]
6-OHDA-induced neurotoxicity in PC12 cells	0.5–5 μM in DMSO, pre-treatment for 2 h	↓ apoptosis; ↑ HO-1, GCLM and GCLC levels; ↑ Nrf2; ↓ Keap1	[89]
6-OHDA-exposed zebrafish	6.25–50 μg/mL in DMSO, pre-treatment for 2 h + incubation for 4 days after 6-OHDA exposure	↑ swimming capacity; ↓ brain tissue damage; ↓ ROS	

↑: upregulation; ↓: downregulation.

#### 2.3.11. Bone Protective Activity

Bones consistently undergo two distinct physiological mechanisms viz. osteoclast-induced bone resorption and osteoblast-induced bone formation. An imbalance between these two leads to the pathogenesis of osteoporosis and rheumatoid arthritis, a chronic disease [92], which is due to the excessive osteoclastic activity in bones that unnoticeably develops until advanced stages with complex pathophysiological conditions. This promisingly occurs among postmenopausal women and the elderly, because of a reduced or non-existent level of estrogen produced in the body [93]. Estrogen acts as a key inhibitor for preventing bone loss. Two recent studies have shown that marine carotenoid fucoxanthin could potentially be utilized as an anti-osteoporotic agent (Table 11). Guo et al. [22] reported that treatment with fucoxanthin in ovariectomized (OVX) rats significantly decreased the body weight gain, uterine index, bone turnover markers, such as Ca, P and OC, and inflammatory markers such as TNF-α, IL-6, and IL-1β, with histopathological evidence confirming the same effects. In addition, fucoxanthin from brown seaweed significantly reduced the osteoclast differentiation and bone resorption ability with the downregulation of the expression of osteoclast-specific markers in osteoclast-like RAW264.7 cells [94]. This study concluded that fucoxanthin had suppressive effects on osteoclast genesis through the modulation of MAP kinase and the Nrf2 signaling pathway (Table 10). Based on recent findings, fucoxanthin offers a promising therapeutic agent in bone protection and can be translated into a clinical study for a precise understanding of its efficacy in human conditions.

#### 2.3.12. Respiratory Protective Activity

Inflammation in the airways of the respiratory system has become an increasing subject of concern worldwide. It is multifactorial and causes morbidity. Recent studies suggest that fucoxanthin has promising effects in alleviating respiratory complications (Table 12). As such, nasal polyps (NPs) are the most common disorder among individuals and are associated with chronic inflammation of nasal mucosa, causing rhinorrhea, headache, and loss of smell. Jung et al. [25] investigated the inhibitory effects of fucoxanthin on myofibroblast differentiation and extracellular matrix production in nasal polyp-derived fibroblast culture, through the modulation of Smad 2/3 and PI3K/Akt/SP-1 signaling pathways. Allergic rhinitis, an inflammatory condition of the inside of nose due to allergen exposure, was repressed when fucoxanthin was given to ovalbumin (OVA)-induced mice in a mechanism of NF-κB/p65 and STAT3 signaling pathways, resulting in a reduced level of IgE and histamine [95]. In a similar study with the asthma model conducted by Yang et al. [32], fucoxanthin effectively decreased ROS and subsequent inflammatory cytokine releases through antioxidant enzyme activity in bronchoalveolar lavage fluid. Recently, fucoxanthin has been isolated from brown algae and assessed for bronchial asthma in two different model systems, inflammatory human tracheal epithelial BEAS-2B cells and OVA-sensitized mice, it significantly decreased monocyte cell adherence, ROS, pro-inflammatory cytokines in vitro, and inhibited hyperresponsiveness, Th2 cytokine production, and eosinophil infiltration in the lungs in vivo, respectively [43]. However, the particulate matter (PM)-induced respiratory disease model was found nowhere in the recent studies; since air pollution and its consequences in respiratory complications are a growing concern globally. Results from the recent studies concluded that dietary fucoxanthin may hold a great promise for the management of respiratory-related diseases, either in acute or chronic conditions effectively.

#### 2.3.13. Skin Protective Activity

Skin acts as the first line of defense in organisms against all external factors, including mechanical damage, radiation, and pathological invasion. The detrimental effects of UV radiation on skin are of growing concern in modern life. It generates ROS with a subsequent increase in inflammatory cytokines, resulting in oxidative DNA damage in keratinocytes [96]. Recent studies have suggested that fucoxanthin from marine algae shows promising protective effects by the restoration of skin, even after radiation (Table 13). Among them, Rodríguez-Luna et al. [24] proposed that fucoxanthin containing cream provided the in vitro reduction of TNF-α, IL-6, ROS, and LDH levels and in vivo reduction of COX-2 and iNOS expressions, as well as an increase in the HO-1 protein level via Nrf-2 pathway. These findings were supported by an in vitro study, where fucoxanthin activated the Nrf2 signaling pathway and epigenetic demethylation of CpG sites in Nrf2 in HepG2-C8 cells and inhibited the TPA-induced transformation of JB6 P+ cells, indicating skin protective effects against cancer [97]. A precise action mechanism of fucoxanthin on skin cells has been clarified by Natsume et al. [98] using an atopic dermatitis model of NC/Nga mice, the topical application of fucoxanthin dramatically inhibited eosinophil infiltration and IL-33 expression, and stimulated the expressions of IL-2, IL-5, IL-13, IL-10, and TGF-β, with regulatory innate lymphoid cells (ILCreg) observed dominantly in keratinocytes and dermal layers. Meanwhile, in the human cell-based model, fucoxanthin significantly decreased the pro-inflammatory cytokines such as IL-6 and IL-8, and increased NATI gene expression for metabolism [39]. All-trans fucoxanthin from brown alga presented excellent photoprotective potential, as characterized by an acceptable level of photodegradation of UVA and UVB with decreased ROS production [34]. Most of the investigators summarize that fucoxanthin’s effects on skin protection are due to the antioxidative and anti-inflammatory mechanisms, as confirmed by in vitro and in vivo findings, indicating the possibility for use in clinical research for pharmaceutical drug development.

#### 2.3.14. Antimicrobial Activity

Fucoxanthin has been gaining profound attention as an antimicrobial agent in recent years (Table 14). In a study, 20 bacterial species were evaluated for the antibacterial activity of fucoxanthin using agar disc-diffusion and micro-dilution methods [26]. It was observed that fucoxanthin was active against Streptococcus agalactiae, Staphylococcus epidermidis, and Staphylococcus aureus in the agar disc diffusion method, and Streptococcus agalactiae for the microdilution method, indicating a good antimicrobial agent against some Gram-positive pathogens. Moreover, fucoxanthin isolated from Undaria pinnatifida can potentially interact with intestinal bacteria for inhibiting the growth of pathogenic bacteria and consequently promoting the growth of beneficial bacteria in mice [99]. Studies implied that fucoxanthin could be used as microbiota-targeted functional food materials; however, cellular and molecular patterns of fucoxanthin interactions with microorganisms are asked for further in-depth research.

#### 2.3.15. Other Bioactivities

Despite aforementioned pharmacological properties of fucoxanthin, as evidenced by in vitro and in vivo observations, a few more studies have recently been documented (Table 15). As evidenced by Jiang et al. [41], fucoxanthin prevented lipopolysaccharide-triggered depressive behavior in mice by decreasing immobility time in forced swimming and tail suspension test and downregulating IL-1β, IL-6, TNF-α, iNOS, and COX-2 levels; this effect was attributed to the modulation of the AMPK-NF-κB signaling pathway. Another study revealed that fucoxanthin can be alleviated ulcerative colitis in the dextran sulfate sodium (DSS)-induced mice model via anti-inflammatory actions [10]. Graves’ orbitopathy is an autoimmune disease characterized by swelling tissues around the eyes; this condition was significantly alleviated in fucoxanthin-administered mice with decreased levels of IL-17, 8-OHdG, and MDA [100]. Fucoxanthin was tested in mice with thyroid injury induced by cadmium-promoted T4, T3, catalase, and APX levels, and inhibited MDA, apoptosis formation, and endoplasmic reticulum stress, because fucoxanthin potentially inhibited ERK1/2 pathway following treatment [101]. Moreover, in vivo findings of dexamethasone-induced skeletal muscle loss in mice could be reversed by the oral administration of fucoxanthin, through the activation of the mTOR pathway and the suppression of the AMPK pathway, in addition to the effect of fat loss [102].

## 3. Pharmacokinetics of Fucoxanthin

To date, a few literature reviews on the various pharmacological properties of fucoxanthin have been published, but a distinct pharmacokinetic profile of fucoxanthin in clinical and pre-clinical levels is not very well documented. The pharmacokinetics of fucoxanthin aims to show how a drug changes after administration through absorption, distribution, metabolism, and excretion (ADME). The absorption capability of fucoxanthin isolated from Laminaria japonica was elucidated after the peroral administration of 160 nmol per mice by Barkia et al. [103]; fucoxanthinol and amarouciaxanthin A were available after 1 h in blood plasma of which Tmax of both metabolites was 4 h and C_max_ for fucoxanthinol was twice as high as amarouciaxanthin A. The metabolism of fucoxanthin (2 mg/kg) to fucoxanthinol was reported to be quicker and found after 5 min post-injection; area under the curve (AUC) of fucoxanthin was 2.5 times higher when compared with fucoxanthinol. Another study conducted after the peroral administration of 65 mg/kg; the quick conversion of fucoxanthin to fucoxanthinol was detected and found in plasma after 0.5 h of administration, Tmax values for fucoxanthin and fucoxanthinol were 7.7 and 11 h, respectively [104]. In studies on the tissue distribution of fucoxanthin and its two metabolites after peroral administration, maximal levels of fucoxanthinol and amrouciaxanthin A were reported in the liver, followed by lung, kidney, heart and spleen, and can be detected in adipose tissue until 72 h after administration, but not more than 24 h for other tissues [105]. The mean distribution volume of fucoxanthin in the blood was lower than that of its metabolite fucoxanthinol (0.7 L/kg versus 8.8 L/kg) after i.v. injection in rats [104]. Fucoxanthin metabolism is accompanied by hydrolysis with digestive enzymes, lipase, and cholesterol esterase in the gastrointestinal tract, resulting in a conversion of fucoxanthinol, which is eventually absorbed in the intestine (Figure 1). Then, it is further converted to amarouciaxanthin A in the liver by short-chain dehydrogenase/reductase (Figure 1) [106]. The conversion of fucoxanthin to fucoxanthinol was quicker when observed in both i.v. and peroral treatment in rats. The elimination of fucoxanthin was 30-fold higher than its metabolites, with fucoxanthinol (T_1/2_ = 4.5 h) and amarouciaxanthin A (T_1/2_ = 6.7 h) after peroral administration of fucoxanthin in mice [105]. Moreover, both metabolites were eliminated quickly from the liver (T_1/2_ = 2.5 h); however, it was much slower in the kidney (T_1/2_= 6.3 and 10.1 for fucoxanthinol and amarouciaxanthin A, respectively). The findings of fucoxanthin and its metabolites for pharmacokinetics supported the synergistic biofunctional activities and can be introduced as a potent drug for pharmaceuticals.

## 4. Safety, Toxicity, and Functional Stability of Fucoxanthin

Fucoxanthin is a naturally occurring carotenoid of brown seaweed and some microalgae which have traditionally been used as popular dietary supplements in East Asian countries for generations. This long traditional usage supports the safety of fucoxanthin upon consumption. Beppu et al. [107] conducted a toxicity study with fucoxanthin purified from seaweed in male and female ICR mice given orally at single dose concentrations of 1000 and 2000 mg/kg. In a subsequent repeat-dose study, 500 and 1000 mg/kg were administered orally for 30 days. Both studies showed no mortalities and abnormalities in the histological observation of different organs; however, total plasma cholesterol was elevated, possibly due to the interference of fucoxanthinol of the fucoxanthin metabolite or species variance that warrants further elucidation. Similar findings were reproduced in rats at doses of 10 and 50 mg/kg/day for 28 days [108]. The safety upon administration of fucoxanthin was characterized by the Ames test, micronucleus assay, and oral toxicity studies in mice and rats, showing no mutagenicity (≤5000 mg/plate), genotoxicity (≤2000 mg/plate), mortality (LD50 > 2000 mg/kg), or abnormalities in appearance of any internal organs (NOAEL > 200 mg/kg/day) [27]. Furthermore, fucoxanthinol did not cause any toxicity or adverse effects in the in vivo experiments, and can be available in human plasma after the ingestion of brown seaweed, especially *Undaria pinnatifida*. Studies on various models suggest that fucoxanthin can be considered a safe pharmacological ingredient [109].

The stability study of fucoxanthin is crucial to ensuring its bio-accessibility and bio-functionality in pharmaceutical development. Factors affecting the degradation of fucoxanthin include high temperature, oxygen, light, heavy metals exposure, enzymatic reactions, and long-term storage [110]. Different studies have been conducted to test the stability of fucoxanthin in terms of free molecule, emulsions, encapsulation and fucoxanthin coated nanoparticle conditions [111]. The chemical behavior of fucoxanthin was evaluated using the in vitro digestibility model, where it was exposed to different molecules with varying pH ranges from 2.2 to 7. It was found that fucoxanthin degraded progressively from simulated stomach condition (10%) and to the ileum (20%) accompanied by a transformation into fucoxanthinol. Considering that fucoxanthin is well-stabilized, the inclusion of emulsifiers could be a promising option to efficiently preserve its therapeutic potentials and chemical features over the storage period. Different emulsifiers were tested for stability, of which whey protein was the best to provide fucoxanthin with better stability during storage, as reported by Ma et al. [112]. However, further studies are needed to characterize fucoxanthin bioavailability using different emulsifiers with carrier oils. Another approach to stabilizing fucoxanthin is the encapsulation technique to prevent the alteration of core chemical structure using various microcapsules such as maltodextrin, hydroxypropyl-β-cyclodextrin, gum arabic, isolated pea protein, whey protein isolate, and gelatin [110]. A study conducted with fucoxanthin encapsulated into casein nanoparticles, and the same but with coated chitosan, was subjected to an in vitro assay of a simulated digestion process, representing its exposure to various enzymes and secreted fluids; nevertheless, nanoparticles containing fucoxanthin successfully transform fucoxanthinol through the gastrointestinal tract [111]. A recent study showed that fucoxanthin@polyvinylpyrrolidone nanoparticles effectively delivered fucoxanthin into human colon cancer cells in vitro, confirming a new and targeted therapeutic approach [113]. However, nano-particle-based fucoxanthin treatment should be introduced in animal models before further translation into clinical trials to evaluate the extent of bioactivity.

## 5. Clinical Perspectives of Fucoxanthin

Many of the aforementioned in vitro and in vivo studies have become reality when conducting clinical trials. As such, following the NIH ClinicalTrial.gov database, five clinical trials using either single fucoxanthin or fucoxanthin-rich extract are currently underway at different phases of trials or considering future enrolling. Moreover, a study on human subjects has reached its end, demonstrating anti-obesity effects in obese individuals administering 3 mg of fucoxanthin capsule, with a good safety profile, confirming its potential use as a pharmaceutical drug [15]. Studies with a clinical stage on the mitigation of liver health (ClinicalTrials.gov identifier: NCT03625284), the improvement of non-alcoholic fatty liver disease (ClinicalTrials.gov identifier: NCT02875392), metabolic syndrome of insulin sensitivity and secretion (ClinicalTrials.gov identifier: NCT03613740; phase 2), body weight management for overweight women (ClinicalTrials.gov identifier: NCT04761406), etc., have yet to publish their results. Moreover, cognitive decline in aged populations is a major challenge in the coming years. With mounting evidence of in vitro and in vivo experiments in the recent past, a clinical study on the neuroprotective potential of fucoxanthin-rich microalgae has been initiated since 2021 and continued to date for the validation of its efficacy for future pharmaceutical application (ClinicalTrials.gov identifier: NCT04832412). Considering recent studies, fucoxanthin can be introduced as a lead drug with multi-targets or a pro-type drug for future pharmaceutical or nutraceutical development.

## 6. Pharmaceutical Prospects of Fucoxanthin

**Figure 3:**
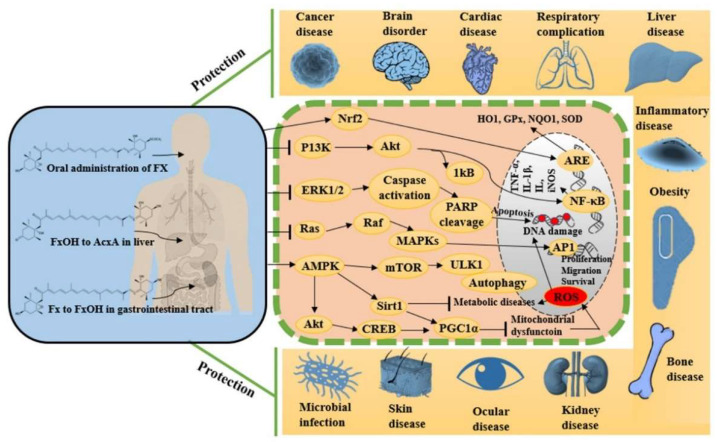
Metabolism of fucoxanthin and its derivatives after administration and their pharmacological modulation in various cellular pathways.

Fucoxanthin is a naturally occurring potent antioxidant molecule with noteworthy and diverse bioactivities. The growing body of scientific in vitro and in vivo evidence leads us to understand the maximization of its utilization in viable and systematic methods for nutraceutical and pharmaceutical development. Since 2017, studies on fucoxanthin have mostly reported promising antioxidant, anti-inflammatory, and anti-apoptotic potentials, together these effects synergistically protect against cancer, neurodegeneration, hyperlipidemia, diabetics, cardiac, liver, kidney, eye and skin diseases, among others. Figure 3 shows the various pharmacological properties of fucoxanthin with possible molecular actions mechanisms by targeting multiple signaling pathways. These effects turn fucoxanthin into further clinical research to evaluate its efficacy in each disease condition before developing it as a therapeutic drug.

Based on the recent scientific literature reviewed in this study, fucoxanthin extracted from edible brown seaweed commercially has been suggested for pharmaceutical or medicinal uses, as it is safe and has no toxicity upon administration. However, the availability of fucoxanthin for industrial uses, unfortunately, is limited, and a chemical process for fucoxanthin synthesis is complex and eventually expensive for endpoint users. To scale up the industrial production, an effective, easy, and low-cost solvent extraction method should be introduced, and standardized to maximize its yield. Thus, seaweed with the highest fucoxanthin content requires more attention to intensive culture rather than depending on natural sources to ensure its year-round availability. The post-extraction stability studies of fucoxanthin recommend that its use at the industry level should be kept at a low temperature and free from heat and light as well, because it is vulnerable to all of these extrinsic factors. Some studies suggest using it in an encapsulated form with either emulsifiers or nanoparticles to preserve its bio-accessibility and bio-functionality while taken as food ingredients or pharmaceutical drugs. During the oral administration of fucoxanthin, two of its metabolites, fucoxanthinol, and amarouciaxanthin A, are readily accessible to the blood plasma, and are located in various organs such as the liver, kidney, and heart for bio-functionalities; those studies were conducted in vivo and human trials [109,110,112]. A wide array of health functional properties of fucoxanthin has been relevant for its future applications both in food supplementation as an active ingredient and pharmaceutical industries as a potent drug.

## 7. Materials and Methods

### 7.1. Literature Search

**Figure 4:**
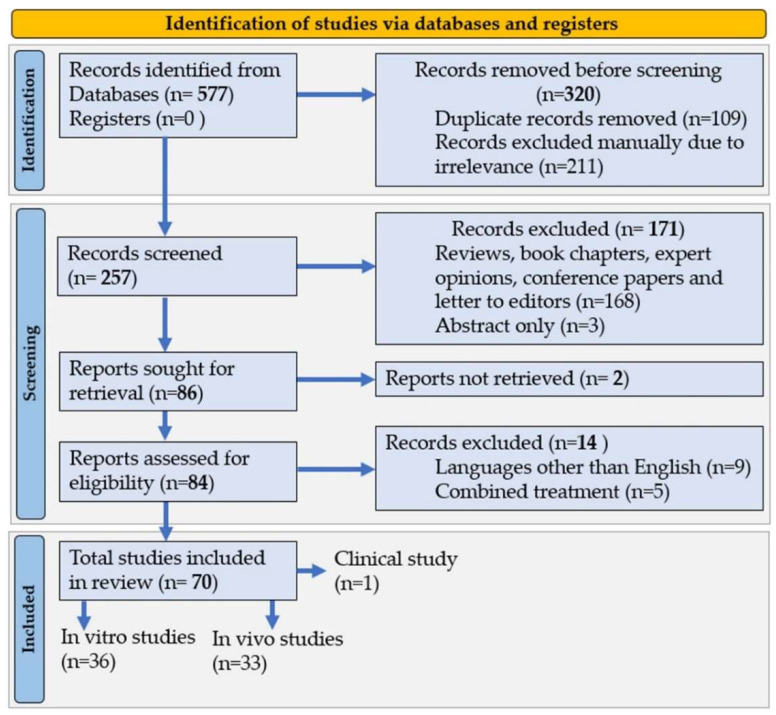
PRISMA 2020 flow diagram for the systematic review of fucoxanthin.

Following the method of Preferred Reporting Items for Systematic Reviews and Meta-Analysis (PRISMA), studies being reviewed were identified, screened, and finally included systematically (Figure 4). Three different search engines such as Scopus, Web of Science and PubMed databases were used to collect the scientific literature. Scientific articles on fucoxanthin published between January 2017 and February 2022 were included in the search. In some cases, for example, in toxicity study, the article number is too limited to conclude. In that case, we also considered previous available studies published before January 2017. The criteria for literature search were “fucoxanthin” and “pharmacology” OR “bioactivities” OR “bio function” OR “biological activities” OR “health” OR “therapeutics” OR “disease” OR “cells” OR “human” OR “animal” OR “organisms”. The total number of the searched article was 577, which was further removed based on duplication of similar titles and keywords (211), and manual exclusion due to irrelevance (109), was reduced to 257.

### 7.2. Selection Criteria

In the second stage of screening, initially, 171 records were excluded from 257, because those were reviews, book chapters, expert opinions, conference papers and letters to editors (168), and only abstracts (3). The remaining records of 86 were used for retrieval and resulted in 84 in the end. The articles were further assessed for exclusion based on a language other than English (9) and studies of combined treatment (5). Finally, 70 articles remained for inclusion in the final stage. Of the 70 articles to be reviewed, those were categorized into in vitro studies (36), in vivo studies (33) and clinical studies (1).

### 7.3. Data Extraction

During the final selection, the research data were extracted from 70 articles on fucoxanthin. Procedures for data collection were set based on the characteristics of the article and the criteria used to assess bioactivities. The following data were extracted (1) year of publication, (2) experimental model (either in vitro or in vivo), (3) treatment (dose range, route of administration and duration), and (4) major outcomes with molecular targets.

## 8. Conclusions

Our review concludes that the structure of fucoxanthin possesses specialized features that permit a variety of pharmacological attributes, including antioxidant and anti-inflammatory activities, providing synergistic protection against neurological, obesity, hepatic, diabetic, kidney, cardiac, skin, respiratory, and microbial diseases. Research trends of fucoxanthin have increased dramatically, especially in the last five years, among which in vivo studies have the majority (45%), followed by animal cells (25%), human cells (24%), microbial (3%), and clinical study (0.75%) model systems. We provide updated knowledge on the molecular pharmacology of fucoxanthin to minimize the research gaps. To gain further insights into molecular pharmacology against disease-specific targets, modern approaches such as network pharmacology and computational techniques need to be employed. Moreover, an organ-specific and active drug delivery system using fucoxanthin together with nanoparticles can be for future research and introduced as a new therapeutic with high efficiency and accuracy for preventing and treating various diseases.

The commercial availability of fucoxanthin is hard to find due to the many challenges associated with its production. The chemical synthesis of fucoxanthin provides a complex process that makes it difficult, and the solvent extraction method from marine sources has yet to be standardized. To ease the production process with simple, rapid, and low-cost techniques, future studies can focus on optimizing the extraction conditions with different green technologies, e.g., supercritical, pressurized liquid, microwave-assisted, ultrasound-assisted, etc., which speed up the sustainable and economic friendly production of fucoxanthin towards commercialization.

Observation suggests that the translation of preclinical results into clinical trials is limited. To facilitate the translational research into human objects, additionally, we highlight the pharmacokinetics, safety, toxicity, functional stability, and clinical perspective of fucoxanthin and its metabolites, suggesting the possibility to be used in nutraceutical and pharmaceutical levels. This systematic review will guide future research on conducting the increased number of clinical trials to validate the synergistic effects of fucoxanthin before developing it as a potent drug of natural origin.

## Figures and Tables

**Table 4:** An updated summary of anti-hyperlipidemic and anti-obesity potentials of fucoxanthin: In vitro and in vivo studies.

Experimental Model (In Vitro/In Vivo)	Treatment (Dose, Route and Duration)	Major Outcomes	Reference
Double-blind placebo-controlled study in mild obese Japanese subjects	1 or 3 mg daily, orally for 4 weeks	↓ relative body weight and BMI and visceral; ↓ fat area and mass	[15]
Fatty acid-induced lipid accumulation in FL83B cells	3–100 μM (purity ≥ 95%) in DMSO, Post-treatment for 24 h	↓ lipid accumulation, lipid peroxidation; ↓ PPARγ and SREBP-1c; ↑ CPT-1 and PPAR-a; ↑ Sirt1 and AMPK	[16]
Hyperlipidemia in diabetic mice	0.2–0.4%/day (purity ≥ 98%) extracted from *Laminaria japonica*, orally for 6 weeks; 0.02% metformin as positive control	↓ plasma insulin and HOMA-IR; ↓ TG and TC levels; ↓ Glucokinase and phosphoenolpyruvate; Carboxykinase; ↑ Glycogen and GLUT4; ↓ GSK3β; ↑ IRS-1, PI3K, p-Akt and p-AMPK	[66]
High-fed diet mice intestine	125 mg/kg b.w. (purity ≥ 95%) extracted from undaria pinnatifida, orally for 4 weeks	Modulation of gut microbiota to exert anti-obesity effects	[64]
HFD-induced obesity mice	100–300 mg/kg b.w., orally for 26 days	↑ Cpt1; Ucp1; ↓ Mest; ↓ body weight gain; ↓ fat content; ↓ weight of white adipose tissue depots and size	[61]
3T3-L1 cells	10–40 μM extracted from Phaeodactylum in DMSO, treatment for 6 days	↓ lipid accumulation; ↓ C/EBPα, PPARγ and UCP1	[62]
High-fat diet-induced mice	0.1 mg/kg b.w. extracted from Phaeodactylum in DW water, orally for 6 weeks	↓ TG level; ↓ lipid droplet numbers and fat globule size ↓ C/EBPα, PPARγ and UCP1	
HFD-fed obese mice	50–100 mg/100 g diet (purity ≥ 93%) extracted from *Undaria pinnatifida*, orally for 12 weeks	↓ body weight gain ↑ HDL-cholesterol level ↓ hepatic steatosis and adipocyte size ↓ IL-6 and TNF-α levels	[17]
HFD-induced obese mice	0.2–0.4% of daily diet, orally for 6 weeks	↓ body weight, TC, TG, LDL-C and HOMA-IR; ↑ HDL-C; ↑ p-IRS-1, IRS-1, PI3 K and p-Akt	[65]
HFD-fed obese mice	0.5 mg/kg b.w. (purity ≥ 95%) extracted from *Padina tetrastromatica*, orally for 5 weeks; orlistat 20 mg/kg as positive control	↓ body weight, TC, TG; ↑ SOD, CAT and GPx; ↑ Akt and UCP-1 ↓ p-Akt, p38 and PPAR-γ	[63]

↑: upregulation; ↓: downregulation.

**Table 5:** An updated summary of antidiabetic activities of fucoxanthin: In vitro and in vivo studies.

Experimental Model (In Vitro/In Vivo)	Treatment (Dose, Route and Duration)	Major Outcomes	Reference
Type-2 diabetic mice	0.2–0.4%/day (purity ≥ 98%) extracted from *Laminaria japonica* in soybean oil, orally for 6 weeks; metformin 0.02% as positive control	↓ body weight and blood glucose; ↓ plasma insulin, HOMA-IR levels and lipid profile; ↑ Glucokinase mRNA: ↓ phosphoenolpyruvate carboxykinase mRNA; ↑ glycogen synthesis: ↑ IRS-1, PI3K, p-Akt and p-AMPK signaling pathways; ↑ PPARα, p-ACC and CPT-1 protein expression	[66]
STZ-and NA-induced diabetic rats	13–65 mg/kg b.w. extracted from *Laminaria japonica*, orally for 4 weeks; rosiglitazone 0.571 mg/kg as positive control	↓ plasma glucose, insulin level and HOMA-IR; ↑ CAT, SOD and GPx; ↓ TNF-α and IL-6; ↑ luteinizing and testosterone hormones	[3]
HG-and 4-HNE-induced diabetic retinopathy in ARPE-19 cells	0.1–0.5 mg/mL, co-treatment for 24–72 h	↓ cell damage; ↓ inflammation response; ↓ apoptosis; ↓ cell adhesion factor protein; ↓ reactive oxygen species; ↑ antioxidant activity	[23]
STZ-and NA-induced type 2 diabetic rats	400 mg/kg b.w. (purity ≥ 54%) extracted from *S. angustifolium*, encapsulated with porous starch, orally for 3 weeks; metformin 50 mg/kg as positive control	↓ weight gain and blood glucose; ↑ plasma insulin; ↓ lipid profile ↑ pancreatic beta cells	[68]
HG-induced GMCs in diabetic nephropathy	2 μM, co-treatment for 24 h	↓ fibronectin and collagen IV expression; ↑ Sirt1/Nrf2 signaling proteins	[67]
STZ-induced diabetic rats	200 mg/kg b.w., orally for 12 weeks	↑ renal function and hypertrophy; ↓ glomerulosclerosis; ↓ fibronectin and collagen IV expression; ↑ Sirt1/Nrf2 signaling proteins; ↑ SOD and HO-1; ↓ malondialdehyde level	

↑: upregulation; ↓: downregulation.

**Table 6:** An updated summary of cardioprotective activities of fucoxanthin: In vitro and in vivo studies.

Experimental Model (In Vitro/In Vivo)	Treatment (Dose, Route and Duration)	Major Outcomes	Reference
Aging C57BL mice	250–500 mg/kg b.w., orally for 28 days	↓ SOS1 and GRB2; ↓ ROS; ↑ GSK3β, CREB and IRS1	[72]
Doxorubicin-induced cardiac dysfunction mice	125–500 mg/kg b.w., intraperitoneally for 4 days	↓ AST, LDH and CKMB	[21]
Doxorubicin-treated neonatal rat cardiomyocytes	50 µM in ddH_2_O, pre-treatment for 24 h	↓ ROS; ↓ Bax, p-ERK, p-JNK, p-p38 and p-p53; ↑ GSH and Bcl-2	
Isoprenaline hydrochloride- induced myocardial infarction rats	50 mg/kg b.w. (purity ≥ 95%), orally for 30 days	↑ SOD, CAT, GPx and GSH; ↓ CKMB, TNF-α, IL-6 and NF-κB	[73]
H_2_O_2_-treated rat valve interstitial cells	0.01–5 mg/mL in ddH_2_O, pre-treatment for 24 h	↓ c-PARP, Caspase 3 and Bax; ↑ Bcl-2; ↓ ROS and Akt/p-Akt	[70]

↑: upregulation; ↓: downregulation.

**Table 7:** An updated summary of hepatoprotective activities of fucoxanthin: In vitro and in vivo studies.

Experimental Model (In Vitro/In Vivo)	Treatment (Dose, Route and Duration)	Major Outcomes	Reference
Fatty acid-induced lipid accumulation in FL83B hepatocytes	3–100 μM (purity ≥ 95%) in DMSO, post-treatment for 24 h	↓ Sterol regulatory element-binding proteins 1c and peroxisome proliferator-activated receptor γ; ↓ Fatty acid synthase expression, acetyl-CoA carboxylase; ↑ Adipose triglyceride lipase and the phosphorylation of hormone-sensitive lipase, p-AMPK	[16]
AA+ iron-induced oxidative stress in HepG2 cells	30 μM, pretreatment for 1 h	↑ Autophagic markers (LC3II and beclin-1), AMPK activation; ↓ p-mTOR; ↑ p-ULK1	[75]
DEN-induced liver carcinoma rats	50 mg/kg b.w., orally for 15 weeks	↑ Body weight, serum albumin, SOD, CAT, GPx, GR; ↓ ALT, AST, ALP, LDH, GGT, serum bilirubin and stress markers	[76]
Alcohol-induced liver injury mice	10–40 mg/kg b.w. in alcohol, orally for 7 days; silibinin 80 mg/kg b.w. orally as positive control	↑ T-AOC, GSH-Px, SOD and CAT; ↓ MDA; ↑ ADH and ALDH; ↓ TNF-α, IL-1β, IL-6, IFN -γ; ↑ Nrf2 protein, NQO1, HO-1 and GCLM; ↓ MyD88, p-IκBα and p-NF-κBp65	[9]

↑: upregulation; ↓: downregulation.

**Table 8:** An updated summary of reno-protective activities of fucoxanthin: In vitro and in vivo studies.

Experimental Model (In Vitro/In Vivo)	Treatment (Dose, Route and Duration)	Major Outcomes	Reference
HG-induced renal fibrosis in mesangial cells	2 µM (purity ≥ 90%), co-treatment for 24 h	↓ Fibronectin, collagen IV and extracellular matrix; ↓ ROS; ↓ Serine-threonine kinase; ↑ Sirt1, FoxO3α and MnSOD	[79]
Ethylene glycol-treated urolithiasis rats	40–80 mg/kg b.w. (purity ≥ 99%) in potable water, orally for 4 weeks	↓ AST, ALT, ALP, GGT and LPO; ↑ SOD, CAT, GPx and GSH	[80]
HG-treated mesangial kidney Mes13 cells	1–2 µM (purity ≥ 98%) in 0.1% DMSO, co-treatment for 5 days	↓ ROS; ↓ Disp2, ATG10 and CYP2E1; ↑ FGF1, WNT7B and Tgfb1i1	[20]
HG-treated glomerular mesangial cells and STZ -induced diabetic rats	N/A	↑ Sirt1, ↑Nrf2, ↑SOD and ↑HO-1	[67]
Cadmium chloride-treated mice	10–50 mg/kg b.w., orally for 14 days; shenfukang tablets orally 50 mg/kg b.w./day for 14 days as positive control	↓ Blood urea nitrogen and KIM-1; ↓ Caspase 3, Caspase 8, Caspase 9, ERK2, NGAL and POD; ↑ SOD, CAT and APX	[8]

↑: upregulation; ↓: downregulation.

**Table 9:** An updated summary of ocular protective activities of fucoxanthin: In vitro and in vivo studies.

Experimental model (In Vitro/In Vivo)	Treatment (Dose, Route and Duration)	Major Outcomes	Reference
UVB-Induced corneal denervation rats	1–10 mg/kg b.w., orally for 6 days	↑ Nrf2 in cornea; ↓ p38 MAP kinase and GFAP-positive neural cells; ↑ nerve innervation ↓ TRPV1 expression in the trigeminal ganglia neurons; ↓ opening the eyes and eye wipe behavior	[42]
High glucose and 4-HNE-induced diabetic retinopathy in ARPE-19 cells	0.1–0.5 mg/mL, co-treatment for 24–72 h	↑ cell viability; ↓ DNA damage; ↑ Nrf2 protein; ↓ apoptosis- related protein expression; ↓ ICAM-1; ↑ occludin and ZO-1 protein expressions; ↓ ROS; ↑ antioxidant activity	[23]
LPS-induced uveitis rats	1–10 mg/kg b.w. in 0.1% DMSO, orally for 7 days	↑ Nrf2 in ocular tissues; ↑ SOD; ↓ MDA; ↓ ocular hypertension; ↓ inflammatory cells and TNF-α; ↓ corneal endothelial disruption	[33]

↑: upregulation; ↓: downregulation.

**Table 11:** An updated summary of bone protective activities of fucoxanthin: In vitro and in vivo studies.

Experimental Model (In Vitro/In Vivo)	Treatment (Dose, Route and Duration)	Major Outcomes	Reference
Ovariectomy-induced osteoporosis rats	20–40 mg/kg b.w., orally for 16 weeks	↓ IL-6, TNF-α and IL-1β; ↑ serum levels of E2 and 1,25(OH)2 D3; ↓ RANKL; ↑ OPG levels; ↑ bone mineral contents and density; ↑ normal bone architecture and trabecular formation in femur;	[22]
sRANKL and/or NF-κB-induced osteoclast-like RAW264.7 cells	1–5 μM, pre-treatment for 4 days	↓ osteoclast differentiation and bone resorption ability; ↓ nuclear factor of activated T cells 1, dendritic cell-specific seven transmembrane protein and matrix metallopeptidase 9; ↓ p38 and ERK; ↑ nuclear translocation of phospho-Nrf2	[94]

↑: upregulation; ↓: downregulation.

**Table 12:** An updated summary of respiratory protective activities of fucoxanthin: In vitro and in vivo studies.

Experimental Model (In Vitro/In Vivo)	Treatment (Dose, Route and Duration)	Major Outcomes	Reference
Nasal polyps-derived fibroblast culture	10–30 µM, treatment for 24 h; TGF-β1 as negative control	↓ α-SMA and Col-1; ↓ collagen gel contraction; ↓ Smad-2/3 and SP-1	[25]
OVA-induced allergic rhinitis mice	N/A	↓ ciliary loss, eosinophil infiltration and MDA; ↑ NF-κB p65; ↓ IκBα phosphorylation; ↓ IL-17A expression; ↓ IgE and histamine	[95]
OVA-induced asthma mice	50 mg/kg b.w., oral treatment	↓ ROS; ↑ antioxidant enzyme activity; ↓inflammatory cytokine markers;	[32]
Inflamed tracheal epithelial BEAS-2B cells	3–30 μM (purity ≥ 95%) in DMSO, pre-treatment for 1 h; TNF-α/IL-4 as negative control	↓ THP-1 cell adherence; ↓ pro-inflammatory cytokines, eotaxin and ROS	[43]
OVA-sensitized mice	10–30 mg/kg b.w. (purity ≥ 95%) in DMSO, intraperitoneally for every 3 days from day 14 to 27; prednisolone as positive control	↓ AHR, goblet cell hyperplasia and eosinophil infiltration; ↓ Th2 cytokine production	

↑: upregulation; ↓: downregulation.

**Table 13:** An updated summary of skin protective activities of fucoxanthin: In vitro and in vivo studies.

Experimental Model (In Vitro/In Vivo)	Treatment (Dose, Route and Duration)	Major Outcomes	Reference
UVB-irradiated HaCaT cells	10–100 μM in 0.1% DMSO, pre-treated for 24 h; dexamethasone as positive reference control	↑ viability; ↓ TNF-α, IL-6; ↓ ROS and LDH production;	[24]
TPA-induced epidermal hyperplasia in mice	200 μg in ethanol of cream formulation/cm2 skin area, topical application for 5 days; β-carotene-cream as positive control	↓ skin edema, epidermal thickness, MPO activity; ↓ COX-2 and iNOS expression; ↑ HO-1 protein	
TPA-induced transformation of JB6 P+ cells	6.25–50 μM in 0.1% DMSO, co-treatment for 3–24 h; 5-aza-deoxycytidine and trichostatin A as positive control	↑ Nrf2 and its downstream genes; ↓ colony formation in JB6 P+ cells; ↓ methylation of the Nrf2 promoter region; ↓ DNMT activity	[97]
Atopic dermatitis Nc/Nga mice	0.1% (purity: 70%) in vaseline, topical application for 5 weeks; 0.1% tacrolimus ointment as positive control	↓ eosinophil infiltration and expression of Il-33; ↑ IL-2, IL-5, IL-13, IL-10 and TGF-β expression; ↑ innate lymphoid cells	[98]
Reconstructed human skin in culture plates	0.5% (*w*/*v*) all-trans-fucoxanthin (purity ≥ 95%) in alkyl benzoate or ethanol, pre-treatment for 15 min; sodium dodecyl sulfate as positive control	↑ viability; ↓IL-6 and IL-8; ↑ NAT1 gene expression	[39]
UVA-and UVB-induced 3T3 mouse fibroblast cells and reconstructed human skin	0.1–100 μg/mL extracted from D. anceps in sunscreen formulation, pre-treatment for 1 h; norfloxacin as positive control	↓ phototoxicity; ↓ acute photoirritation potential; ↓ ROS	[34]

↑: upregulation; ↓: downregulation.

**Table 14:** An updated summary of antimicrobial activities of fucoxanthin: In vitro and in vivo studies.

Experimental Model (In Vitro/In Vivo)	Treatment (Dose, Route and Duration)	Major Outcomes	Reference
Agar disc-diffusion	15.6–1000 μg/mL (purity ≥ 95%) in 20% water solution of DMSO, incubation for 18 h and anaerobes for 2 h	*Streptococcus agalactiae* (mean ZOI 12.2 mm), Staphylococcus epidermidis (mean ZOI 11.2 mm) and Staphylococcus aureus (mean ZOI 11.0 mm)	[26]
Micro-dilution test	15.6–1000 μg/mL, incubation for 24 h	*Streptococcus agalactiae* with minimal inhibitory concentration of 62.5 μg/mL	
Agar disc-diffusion	4.25 mg/mL (purity ≥ 82.70%) extracted from *Undaria pinnatifida* in dehydrated alcohol, incubation for 24 h; chloramphenicol as positive control	↓ Gram-positive pathogenic bacteria	[99]
Gut microbiome of mice cultured in brain heart infusion broth anaerobically	0.025–0.1 mg/mL, incubation for 48 h	↑ intestinal beneficial microbes	

↑: upregulation; ↓: downregulation.

**Table 15:** An updated summary of other bioactivities of fucoxanthin: In vitro and in vivo studies.

Experimental Model (In Vitro/In Vivo)	Treatment (Dose, Route and Duration)	Major Outcomes	Reference
LPS-induced behavioral defects mice	50–200 mg/kg b.w. (purity ≥ 95.0%) in 0.5% sodium carboxymethylcellulose, orally for 7 days	↓ immobility time in forced swimming and tail suspension test; ↓ IL-1β, IL-6 and TNF-α; ↓ iNOS and COX-2	[41]
DSS-induced colitis mice	50–100 mg/kg b.w., orally for 7 days	↓ body weight loss; ↓ increase of disease activity index and colon shortening; ↓ colon histological damages; ↓ colonic PGE2, COX-2 and NF-κB levels	[10]
Graves’ orbitopathy-induced mice	50 mg/kg b.w., orally for 4 weeks	↓ mRNA expression of IL-17 ↓ 8-OHdG and MDA	[100]
CdCl_2_-induced thyroid damage mice	10–50 mg/kg b.w., orally for 14 days; thyroid tablets 50 mg/kg b.w. as positive control	↑ T4, T3, catalase and APX levels; ↓ MDA; ↑ apoptosis inhibition; ↓ endoplasmic reticulum stress	[101]
Dexamethasone-induced skeletal muscle loss mice	0.2% of daily diet, orally for 14 days	↓ muscle atrophy, visceral fat mass and muscle lipid peroxidation; ↑ phosphorylation of mTOR; ↓ activation of AMPK	[102]

↑: upregulation; ↓: downregulation.

## Data Availability

Data supporting the reported results are available upon request.

## Abbreviations

AA: Arachidonic acid, ADH: Alcohol dehydrogenase, AHR: Airway hyper-responsiveness. Akt: protein kinase B, ALDH: Aldehyde dehydrogenase, ALP: Alkaline phosphatase, ALT: alanine aminotransferase, AMPK: 5′ adenosine monophosphate-activated protein kinase, AOM: Azoxymethane, AP-1: transcription factor, APAF: Apoptotic peptidase activating factor, APX: Ascorbate peroxidase, ARE: Nuclear antioxidant response element, Arg1: Arginase 1, ARPE-19: Human retinal epithelial cells, ASC: Apoptosis-associated speck-like protein containing a CARD, AST: Aminotransferase, ATG10: Autophagy-related 10, ATP: Adenosine triphosphate, Aβ1-42: Amyloid β-Protein 1-42, B.W.: body weight, BAX: BCL2 associated X, Bcl-2: B-cell lymphoma 2, BDNF: Brain derived neurotrophic factor, BEAS-2B cells: Human bronchial epithelial cells, BID: A Bax-like BH3 protein, BMDCs: Bone marrow-derived dendritic cells, BMDMs: Bone marrow-derived macrophases, BTLA: B and T lymphocyte associated, BV2: Microglial cells, C/EBPα: CCAAT/Enhancer-binding Protein α, Caco-2 cells: Human colon epithelial cancer cell line, CAT: Catalase, CCL21: C-C Motif chemokine ligand 21, CCL5: C-C Motif Chemokine Ligand 5, Ccr1: C-C motif chemokine receptor 1, CCR7: CC-chemokine receptor 7, CCSCs: Colorectal cancer stem cells, CDAHFD: Choline-deficient L-amino acid-defined high fat diet, ChAT: Choline acetyltransferase, CKMB: Creatine kinase-MB, Col-1: Collagen type I, COX-2: Cyclooxygenase-2, c-PARP: Cleaved poly ADP-ribose polymerase, CPT-1: Carnitine palmitoyltransferase I, CPT1a: Carnitine Palmitoyltransferase 1A, CREB: cAMP-response element binding protein, Csps: Colonospheres, CYCS: Cytochrome C, somatic, VEGF: Vascular endothelial growth factor, CYP2E1: Cytochrome P450 2E1, DEN: Diethylnitrosamine, Disp2: Dispatched RND transporter family member 2, DNMT: DNA methyltransferase, DPPH: 2,2-diphenyl-1-picrylhydrazyl, DSS: Dextran sulfate sodium, ECH-associated protein 1.ERK: Extracellular signal-regulated kinase, FGF1: Fibroblast growth factor 1, FL83B cells: Hepatocyte cell line, FoxO3α: Forkhead box O3α, GCLC: Glutamate-cysteine ligase catalytic subunit, GCLM: Glutamate-cysteine ligase modifier, GFAP: Glial fibrillary acidic protein, GGT: Gamma-glutamyl transferase, GLUT4: Glucose transporter type 4, GMCs: glomerular mesangial cells, GPx: Glutathione peroxidase, GR: Glutathione reductase, GRB2: Growth factor receptor-bound protein 2, GSH: Glutathione, GSH-Px: glutathione peroxidase, GSK3β: Glycogen synthase kinase 3 beta, GSSG: Glutathione disulfide, H/R: Hypoxia/reoxygenation, H_2_O_2_: Hydrogen peroxide, HaCaT: keratinocyte cell line, HDL: High-density lipoprotein, HeLa cells: Human epithelial carcinoma cells, HepG2 cells: Hepatocyte carcinoma, HFD: High-fed diet, HG: High glucose, HIST1H3D: Histone H3.1 gene, HO-1: Heme oxygenase-1, HOMA-IR: Homeostatic model assessment-insulin resistance, I/R: ischemic/reperfusion, ICAM-1: Intercellular adhesion molecule-1, ICV: Intracerebroventricular, IFN-γ: Interferon gamma, IgE: Immunoglobulin E, IL: interleukin, iNOS: Inducible NO synthase, IRS1: Insulin receptor substrate 1, IRS-1: Insulin receptor substrate 1, IκBα: Nuclear factor of kappa light polypeptide gene enhancer in B-cells inhibitor alpha, JNK: c-Jun N-terminal kinases, Keap1: Kelch-like, KIM-1: Kidney injury molecule-1, LDH: Lactate dehydrogenase, LDL-C: low-density lipoprotein-cholesterol, LPO: Lipid peroxidation, LPS: Lipopolysaccharide, LX-2: human hepatic stellate cell line; MAP1LC3: Microtubule-associated protein light chain 3II, MAPK: Mitogen-activated protein kinase, MCAO: Middle cerebral artery occlusion, Mcl-1: Myeloid leukemia 1, MCP-1: Monocyte chemoattractant protein-1, MDA: Malondialdehyde, micro-LVD: micro-lymphatic vessel density, MMP-2: Matrix metalloproteinase-2, MnSOD: Manganese superoxide dismutase, MPO: Myeloperoxidase, MyD88: Myeloid differentiation primary response 88, N.A.: Not available. NA: Nicotinamide, NASH: Nonalcoholic steatohepatitis, NAT1: N-Acetyltransferase 1, NF-κB: Nuclear factor kappa B, NGAL: Neutrophil gelatinase-associated lipocalin, *NLRP3: NLR family pyrin domain containing 3*, NO: Nitric oxide, NOD-SCID: Nonobese diabetic-severe combined immunodeficiency, NQO1: Nicotinamide quinone oxidoreductase 1, Nrf2: Nuclear factor erythrocyte-2-related factor 2, OGD/R: Oxygen glucose deprivation/re-oxygenation, OPG: Osteoprotegerin, OVA: Ovalbumin, p38 MAP: p38 mitogen-activated protein, p53: tumor protein p53, p-ACC: Phospho-acetyl-CoA carboxylase, pAkt: phosphorylated Akt, p-AMPK: Phospho- AMP-activated protein kinase; PARP: poly(ADP-ribose) polymerase-1, PC12 cells: Pheochromocytoma cells, PCNA: Proliferating cell nuclear antigen, pERK: Phospho-extracellular signal-regulated kinase, pFAK: Phospho-focal adhesion kinase, PGE2: Prostaglandin E2, pGSK3*β: Phospho glycogen synthase kinase 3 beta*, PI3K: Phosphoinositide 3-kinase, p-IRS-1: Phospho-insulin receptor substrate 1,p-IκBα: Phospho-nuclear factor of kappa light polypeptide gene enhancer in B-cells inhibitor, alpha, p-JNK: Phospho-c-Jun N-terminal kinases, PLGA-PEGFuc: Poly lactic-co-glycolic acid-block-polyethylene glycol loaded fucoxanthin, PM: Particulate matter, p-mTOR: Phospho-mammalian target of rapamycin, p-NF-κBp65: Phospho-NF-κB p65, POD: Peroxidase, p-p53: Phospho-tumor protein p53, PPAR γ: Peroxisome proliferator- activated receptor gamma, PPARα: Peroxisome proliferator-activated receptor alpha, PPARβ/δ: Peroxisome proliferator-activated receptor beta/δ, PPARγ: proliferator-activated receptor γ, pSmad2: Phosphorylated Smad 2, p-STAT: Phospho-Stat, p-ULK1: Phospho-Unc-51-like kinase 1, RANKL: Receptor activator of nuclear factor kappa-Β ligand, RAW 264.7 cells: Murine macrophage cells, Rho A: Ras homolog family member A, ROS: Reactive oxygen species, SH-SY5Y Cells: Neuroblastoma cell line, Sirt1: Silent information regulator T1, SOD: Superoxide dismutase, SOS1: Son of sevenless homolog 1, SP-1: Signal protein-1,sRANKL: Soluble RANK ligand, SREBP-1c: Sterol regulatory element-binding protein 1, STAT: Signal transducer and activator of transcription, STZ: streptozotocin, T3: Triiodothyronine, T4: Thyroxine, T-AOC: Total antioxidant capacity, TC: Total cholesterol, TG: Triglyceride, Tgfb1i1: Transforming growth factor β-1-induced transcript 1, TGFβ: Transforming growth factor β, THP-1: Human monocytic cells, TIMP-2: Tissue inhibitor of metalloproteinases 2, TNF-α: Tumor necrosis factor-α, TPA: 12-O-tetradecanoylphorbol-13-acetate, TRPV1: Transient receptor potential vanilloid type 1, Ucp1: Uncoupling protein 1, UV: Ultraviolet, UVA: Ultraviolet A, WNT7B: Wnt family member 7B, ZO-1: Tight junction protein 1, αSMA: Smooth muscle alpha-actin,3T3-L1: adipocytes cell line, 4-HNE: 4-hydroxynonenal, 6-OHDA: 6-hydroxydopamine, 8-OHdG: 8-hydroxy-2′ –deoxyguanosine, ZOI: Zone of inhibition.
